# The Diadenylate Cyclase CdaA Is Critical for Borrelia turicatae Virulence and Physiology

**DOI:** 10.1128/IAI.00787-20

**Published:** 2021-05-17

**Authors:** Clay D. Jackson-Litteken, C. Tyler Ratliff, Alexander R. Kneubehl, Cheta Siletti, Lindsay Pack, Renny Lan, TuAnh N. Huynh, Job E. Lopez, Jon S. Blevins

**Affiliations:** a Department of Microbiology and Immunology, University of Arkansas for Medical Sciences, Little Rock, Arkansas, USA; b Section of Tropical Medicine, Department of Pediatrics, Baylor College of Medicine and Texas Children's Hospital, Houston, Texas, USA; c Food Science Department, University of Wisconsin-Madison, Madison, Wisconsin, USA; d Arkansas Children's Nutrition Center, Department of Pediatrics, University of Arkansas for Medical Sciences, Little Rock, Arkansas, USA; University of Pennsylvania

**Keywords:** *Borrelia*, relapsing fever, TBRF, tick-borne relapsing fever, c-di-AMP, CdaA, diadenylate cyclase, second messenger, pathogenesis, cyclic-di-AMP, dinucleotide second messenger, tick-borne pathogens

## Abstract

Relapsing fever (RF), caused by spirochetes of the genus *Borrelia*, is a globally distributed, vector-borne disease with high prevalence in developing countries. To date, signaling pathways required for infection and virulence of RF *Borrelia* spirochetes are unknown. Cyclic di-AMP (c-di-AMP), synthesized by diadenylate cyclases (DACs), is a second messenger predominantly found in Gram-positive organisms that is linked to virulence and essential physiological processes. Although *Borrelia* is Gram-negative, it encodes one DAC (CdaA), and its importance remains undefined. To investigate the contribution of c-di-AMP signaling in the RF bacterium Borrelia turicatae, a *cdaA* mutant was generated. The mutant was significantly attenuated during murine infection, and genetic complementation reversed this phenotype. Because c-di-AMP is essential for viability in many bacteria, whole-genome sequencing was performed on *cdaA* mutants, and single-nucleotide polymorphisms identified potential suppressor mutations. Additionally, conditional mutation of *cdaA* confirmed that CdaA is important for normal growth and physiology. Interestingly, mutation of *cdaA* did not affect expression of homologs of virulence regulators whose levels are impacted by c-di-AMP signaling in the Lyme disease bacterium Borrelia burgdorferi. Finally, the *cdaA* mutant had a significant growth defect when grown with salts, at decreased osmolarity, and without pyruvate. While the salt treatment phenotype was not reversed by genetic complementation, possibly due to suppressor mutations, growth defects at decreased osmolarity and in media lacking pyruvate could be attributed directly to *cdaA* inactivation. Overall, these results indicate CdaA is critical for B. turicatae pathogenesis and link c-di-AMP to osmoregulation and central metabolism in RF spirochetes.

## INTRODUCTION

Relapsing fever (RF) is a globally distributed, vector-borne disease caused by *Borrelia* spirochetes (1–4). RF is characterized by repeated bouts of high-level bacteremia (up to 10^8^ bacteria/ml of blood) that coincide with recurrent cycles of severe febrile illness along with nondescript symptoms such as nausea, myalgia, and chills (4–9). In addition to fever, infection with RF spirochetes is associated with other serious complications, including meningitis, myocarditis, acute respiratory distress syndrome, and perinatal mortality (5, 10–12). RF can be generally divided into two categories defined by the vector that transmits the bacterium, tick-borne relapsing fever (TBRF) and louse-borne relapsing fever (LBRF). Although TBRF is likely an underreported disease in the Americas and Europe, it is a significant cause of illness in many African countries (3–6). Accordingly, TBRF has been reported as the top cause of bacterial infection in Senegal, the most common cause of fever in rural Zaire, and a top 10 cause of death in children under 5 in Tanzania (13–15). TBRF is an endemic disease limited by the distribution of appropriate tick vectors for specific TBRF species, but LBRF has been termed epidemic RF due to its transmission by the human body louse. Because these latter vectors are not restricted geographically, reproduce rapidly, and feed up to five times a day, they are typically associated with larger outbreaks (16–22). In fact, from 1999 to 2000, an outbreak of LBRF in Sudan resulted in 20,000 cases and 2,000 deaths, and, more recently, LBRF has been reported in African refugee camps across Europe (22–29). Although RF remains a significant global public health issue, no literature exists regarding the regulatory pathways required for pathogenesis of the causative *Borrelia* spirochetes.

Nucleotide second messengers are signaling molecules utilized by bacteria to respond to changing environmental conditions (30, 31). Cyclic dimeric AMP (c-di-AMP), specifically, is a nucleotide second messenger predominantly found in Gram-positive bacteria (32, 33). c-di-AMP signaling pathways consist of three general components, (i) diadenylate cyclases (DACs), enzymes which synthesize c-di-AMP from ATP or ADP; (ii) c-di-AMP phosphodiesterases (PDEs), enzymes which degrade c-di-AMP into pApA or AMP; and (iii) receptors or effectors that, when bound to c-di-AMP, exhibit a change in structure or function (32–35). c-di-AMP-mediated signaling has been implicated in the bacterial response to several stresses, including low pH, β-lactam antibiotics, heat shock, reactive oxygen species (ROS), and altered nutrient availability (36–57), but the most well-characterized function of c-di-AMP is its role in regulation of the osmotic response (58). In fact, c-di-AMP impacts osmoregulation at transcriptional, posttranscriptional, and posttranslational levels (34, 58, 59). Likely due to its extensive role in osmoregulation, c-di-AMP is the only known second messenger that is required for *in vitro* viability of numerous bacteria under standard culture conditions (34, 40, 56, 60–63). Conversely, accumulation of c-di-AMP is also toxic in many of these same bacteria, which has led to c-di-AMP being termed an “essential poison” (34, 35, 64–69). Despite this essential role, there are also several bacteria (e.g., Streptococcus mutans, Mycobacterium tuberculosis, Streptococcus pyogenes, and Synechococcus elongatus) in which c-di-AMP is dispensable for *in vitro* growth (41, 44, 53, 70).

Given the role of c-di-AMP in regulating key physiological functions, it is not surprising that control of c-di-AMP levels is important for the virulence of many bacteria. Specifically, inactivation of *dac* results in attenuation of S. pyogenes and Listeria monocytogenes, while infection is attenuated in *pde* mutants of S. pyogenes, Streptococcus pneumoniae, Streptococcus suis, Bacillus anthracis, L. monocytogenes, and M. tuberculosis (44, 45, 54, 67–71). The attenuated infection phenotypes of c-di-AMP pathway mutants are at least partially explained by the critical role of this second messenger in maintenance of normal bacterial physiology, but c-di-AMP appears to also play direct roles in regulation of virulence-associated genes and phenotypes. In S. pyogenes and S. suis, mutation of genes encoding DACs or c-di-AMP PDEs results in reduced expression of numerous virulence factors, and mutation of B. anthracis c-di-AMP PDEs leads to decreased expression of toxins and S-layer proteins (44, 69, 71). Moreover, c-di-AMP regulates biofilm phenotypes of several bacterial pathogens, with most studies correlating increased cytoplasmic c-di-AMP with increased biofilm formation (44, 46, 69, 72–76). The contribution of this second messenger to both physiology and virulence indicates that c-di-AMP levels impact multiple distinct regulatory pathways and highlights the importance and complexity of c-di-AMP signaling in bacteria.

Although c-di-AMP signaling pathways are predominantly found in Gram-positive bacteria, *Borrelia* spirochetes, which are Gram-negative, encode a single DAC (CdaA) and a single c-di-AMP PDE (DhhP) (77). Only two studies have investigated c-di-AMP-dependent signaling in *Borrelia*, and both studies examined the pathway in the context of the Lyme disease (LD) bacterium Borrelia burgdorferi (64, 78). Ye et al. used a conditional mutational strategy to demonstrate that DhhP is required for *in vitro* viability and infection (64). Reduced *dhhP* expression also led to (i) increased levels of intracellular c-di-AMP, (ii) significantly elongated morphology, and (iii) decreased gene expression and protein production of the virulence regulators BosR and RpoS (64). In the second study, Savage et al. showed that overexpression of *cdaA in vitro* led to no identifiable changes in expression of several known regulators and virulence factors (78). Although overexpression of *cdaA* led to an increase in CdaA levels, no increase in intracellular c-di-AMP was observed. These studies have provided insight into functions and regulation of c-di-AMP signaling in *Borrelia*, but several questions remain. Specifically, the physiological role of CdaA has yet to be defined in any *Borrelia* species. Additionally, given the evolutionary divergence, unique pathologies, and distinct enzootic cycles of LD and RF spirochetes, it is possible that c-di-AMP could have unique functions in these two groups of *Borrelia* (3, 4, 79–81).

Herein, we aimed to investigate the role of CdaA in the TBRF spirochete Borrelia turicatae. Using a murine model of RF, we demonstrated that *cdaA* mutants are significantly attenuated and that this phenotype is reversible by genetic complementation. Next, due to the essentiality of c-di-AMP for *in vitro* viability in many bacterial systems, whole-genome sequencing (WGS) was performed on independently generated *cdaA* mutants to identify potential suppressor mutations. These analyses revealed single-nucleotide polymorphisms (SNPs) in genes potentially involved in membrane transport, metabolism, and translation. A conditional *cdaA* mutant was then used to demonstrate that depletion of CdaA is detrimental to bacterial growth and physiology, supporting the hypothesis that compensatory suppressor mutations are required for normal bacterial physiology *in vitro* upon c*daA* mutation. We next investigated phenotypes associated with *cdaA* mutation *in vitro* to identify potential reasons for the infection defect. *cdaA* mutation had no effect on *bosR* or *rpoS* expression or protein production, suggesting that c-di-AMP-mediated regulation may differ between LD and RF spirochetes. Interestingly, the *cdaA* mutant exhibited significant growth defects when treated with salt, cultured at decreased osmolarity, or grown in media lacking pyruvate. The salt treatment phenotype of the *cdaA* mutant was not reversed by genetic complementation, possibly due to suppressor mutations, but growth defects at decreased osmolarity and in media lacking pyruvate could be attributed directly to mutation of *cdaA*. Overall, these data link c-di-AMP signaling to virulence, osmoregulation, and central metabolism in RF spirochetes.

## RESULTS

### Generation and complementation of the BtΔ*cdaA* mutant.

**FIG 1:**
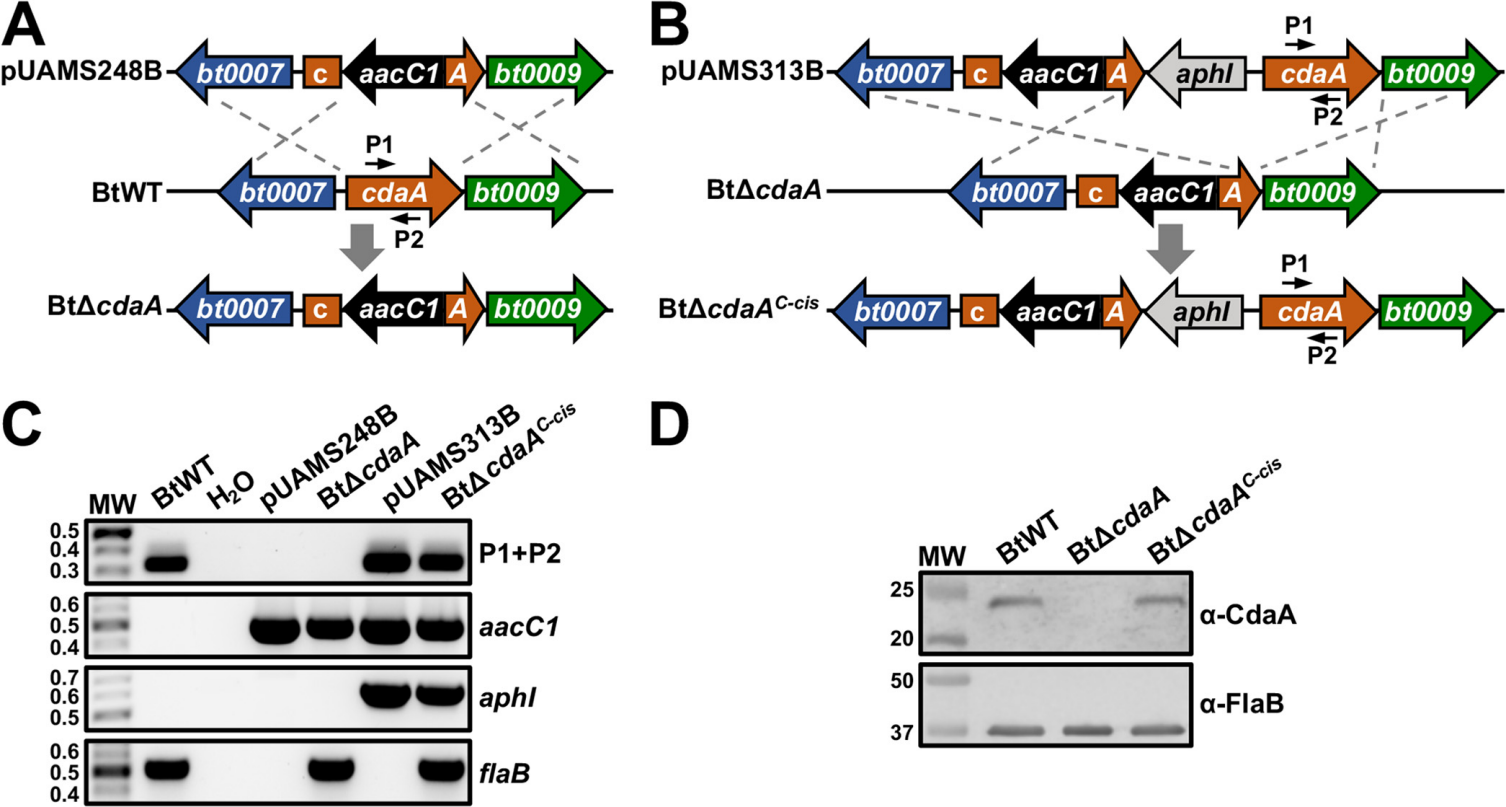
Generation and confirmation of the BtΔ*cdaA* mutant. (A) Generation of the BtΔ*cdaA* mutant. A segment of the *cdaA* ORF was replaced in BtWT with an *aacC1* resistance cassette using allelic exchange mutagenesis to generate BtΔ*cdaA*. Shown are relevant regions of the pUAMS248B mutational construct and BtWT and BtΔ*cdaA* chromosomes. Numbered arrows represent approximate locations of primers used in panel C. (B) Complementation of BtΔ*cdaA*. An *aphI* resistance cassette and *cdaA* with the putative promoter region were inserted into the BtΔ*cdaA* chromosome adjacent to the site of mutation, creating the BtΔ*cdaA^C-cis^* complement. Shown are relevant regions of the pUAMS313B complementation construct and BtΔ*cdaA* and BtΔ*cdaA^C-cis^* chromosomes. Numbered arrows represent approximate locations of primers used in panel C. (C) Genotypic confirmation of BtΔ*cdaA* and BtΔ*cdaA^C-cis^*. PCRs were performed with BtWT, BtΔ*cdaA*, and BtΔ*cdaA^C-cis^* to amplify internal regions of *cdaA* (P1+P2; 328 bp), *aacC1* (489 bp), *aphI* (624 bp), and *flaB* (519 bp). The mutational (pUAMS248B) and complementation (pUAMS313B) constructs were included as positive controls, and reactions with no template (H_2_O) served as a contamination control. MW denotes the DNA standard, and numbers to the left indicate molecular weight in kb. (D) Immunoblot confirmation of BtΔ*cdaA* and BtΔ*cdaA^C-cis^*. Whole-cell lysates of late-exponential-phase BtWT, BtΔ*cdaA*, and BtΔ*cdaA^C-cis^* were separated by SDS-PAGE and transferred to a nitrocellulose membrane. Membranes were then probed with antiserum or antibody against CdaA or FlaB, respectively. Antiserum/antibodies used to detect the respective proteins are indicated to the right. MW denotes the protein standard, and numbers to the left indicate molecular weight in kDa. Two biological replicates were performed, yielding similar results, and a representative blot from one replicate is shown.

c-di-AMP-dependent signaling is important for the virulence of several bacteria (44, 45, 54, 67–71). The chromosomally encoded protein BT0008, referred to as CdaA herein, is predicted to be the only DAC in B. turicatae, and homologs are encoded in all *Borrelia* spirochetes (33, 34, 64, 77, 78). B. turicatae CdaA is a 258-amino-acid, 29.02-kDa protein with a predicted pI of 7.74. CdaA is annotated as a TIGR00159 family protein, and the NCBI Conserved Domain Database identified amino acids 49 to 258 as belonging to the DisA_N superfamily (E value = 1.52e-83), both of which are consistent with CdaA homologs in other bacteria (82–84). Additionally, TMHMM-2.0 predicted CdaA to have three transmembrane domains (amino acids 15 to 32, 39 to 61, and 66 to 85) close to the N terminus (85, 86). The N terminus and C terminus would be located in the periplasm and cytoplasm, respectively, which is characteristic of CdaA proteins (34, 87). Finally, as is the case with other CdaA proteins, two probable coiled-coil domains were identified within the DisA_N superfamily domain (amino acids 109 to 149 and 213 to 251) by Waggawagga coiled-coil prediction analyses (34, 88). To determine the contribution of the B. turicatae *cdaA* homolog during mammalian infection, allelic exchange mutagenesis was used to replace an internal region of the open reading frame (ORF) in wild-type B. turicatae (BtWT) with an *aacC1* gentamicin resistance cassette, generating BtΔ*cdaA* (Fig. 1A). Genetic complementation of BtΔ*cdaA* was achieved by inserting an *aphI* kanamycin resistance cassette and a copy of *cdaA* with the putative promoter region into the BtΔ*cdaA* chromosome adjacent to the site of mutagenesis, creating the BtΔ*cdaA^C-cis^* strain (Fig. 1B). Genotypic confirmation of BtΔ*cdaA* and BtΔ*cdaA^C-cis^* was performed using PCR specific for internal regions of *cdaA*, *aacC1*, and *aphI* genes (Fig. 1C). PCR to amplify a region of the flagellin (*flaB*) gene was also performed as an amplification control. PCR for *cdaA* only produced amplicons in BtWT and BtΔ*cdaA^C-cis^*. In addition, PCRs specific for the resistance markers failed to yield amplicons with BtWT, whereas BtΔ*cdaA* and BtΔ*cdaA^C-cis^* were positive for the *aacC1* gene and *aphI* gene, respectively. Finally, PCR for *flaB* resulted in amplicons of the expected size for all strains. Immunoblot analyses were then performed to assess CdaA production in BtWT, BtΔ*cdaA*, and BtΔ*cdaA^C-cis^* (Fig. 1D). As expected, CdaA was detected in BtWT and BtΔ*cdaA^C-cis^* but undetectable in BtΔ*cdaA*. Importantly, levels of FlaB, which was included as a loading control, were consistent across strains. In all, these results indicate successful mutation of *cdaA* and genetic complementation in BtΔ*cdaA* and BtΔ*cdaA^C-cis^*, respectively.

### The BtΔ*cdaA* mutant is significantly attenuated in a murine needle-challenge model of RF.

**FIG 2:**
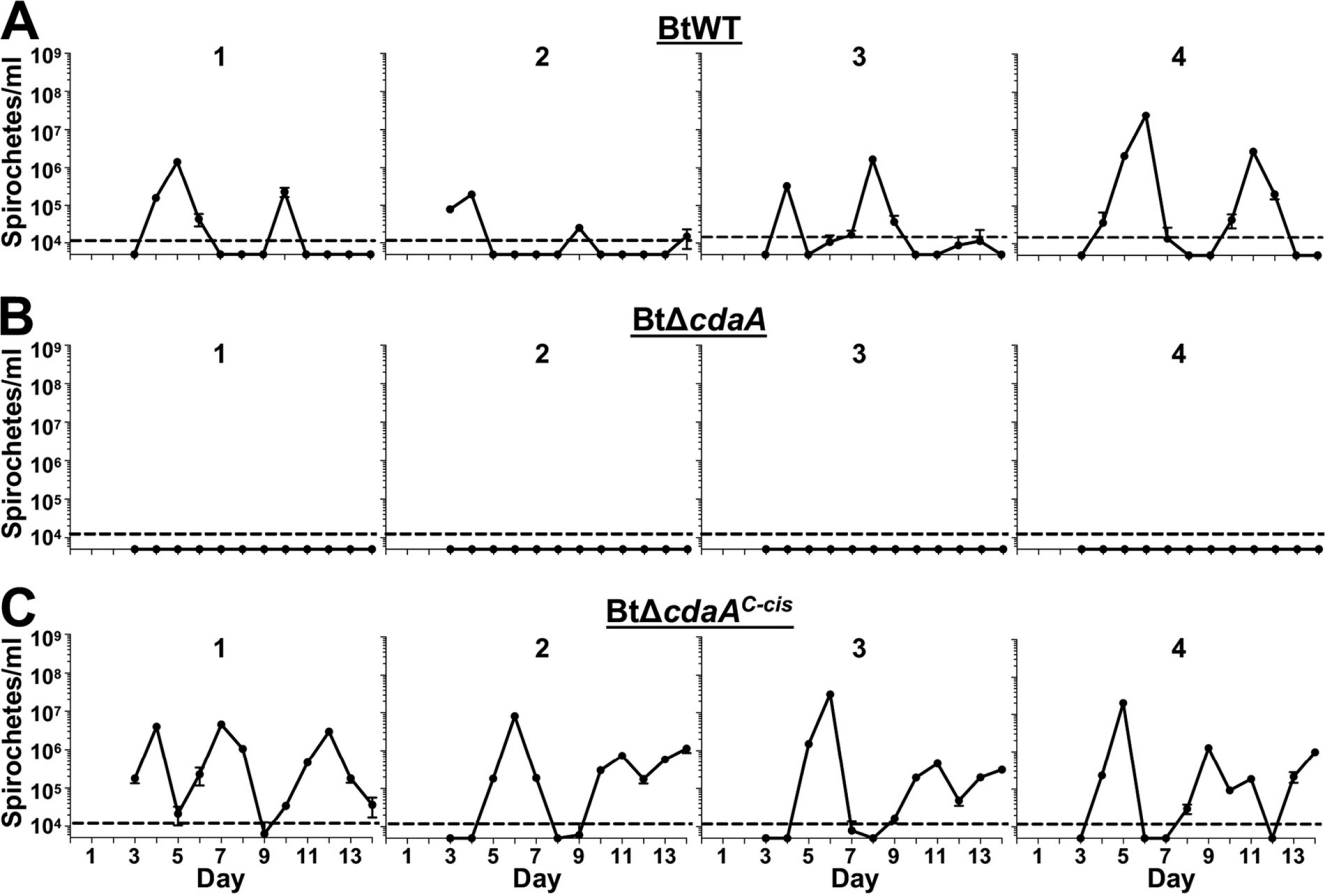
Murine infection phenotypes of BtWT, BtΔ*cdaA*, and BtΔ*cdaA^C-cis^* strains. Groups of four mice were intradermally inoculated with 10^2^ spirochetes of BtWT (A), BtΔ*cdaA* (B), or BtΔ*cdaA^C-cis^* (C). On days 3 to 14 postinfection, bacterial levels in the bloodstream were quantified by qPCR. Numbers above the graphs indicate individual mice in each experimental group, and error bars represent standard error of the mean (SEM). The dashed line indicates the limit of detection (LOD) for this assay (10^4^ spirochetes/ml).

Given the importance of c-di-AMP and DACs for virulence of other bacteria, we hypothesized that CdaA would be required for mammalian infection (44, 54). To test this hypothesis, a murine model of RF was used in which groups of four mice were intradermally needle inoculated with 10^2^ BtWT, BtΔ*cdaA*, or BtΔ*cdaA^C-cis^* bacteria (Fig. 2). On days 3 to 14 postinfection, blood samples were taken, and bacteremia was measured by quantitative PCR (qPCR). As expected, all four mice infected with BtWT and BtΔ*cdaA^C-cis^* experienced recurring bouts of spirochetemia, with the first peak occurring between days 4 to 6 and maximum bloodstream burden ranging from 10^6^ to 10^8^ bacteria/ml of blood (Fig. 2A and C). Conversely, mice infected with BtΔ*cdaA* failed to reach detectable levels of spirochetemia by qPCR on any day postinfection (Fig. 2B).

Because the limit of detection (LOD) with our qPCR is 10^4^ bacteria/ml of blood, it was possible that BtΔ*cdaA* bacteria are still infectious but failed to reach detectable levels (89). Therefore, we also attempted to culture bacteria out of the bloodstream of infected mice during this experiment, which is predicted to provide an LOD of 4 × 10^2^ live bacteria/ml (see Materials and Methods). As expected, days when BtWT- and BtΔ*cdaA^C-cis^*-infected mice were positive by blood culture largely overlapped with days when the mice were positive by qPCR (data not shown). Alternatively, bacteria could not be cultured from the blood of BtΔ*cdaA*-infected mice on any day postinfection (data not shown). As another metric to evaluate infection, mice were also screened for seroconversion at 14 days postinfection against BtWT whole-cell lysates; all BtWT- and BtΔ*cdaA^C-cis^*-infected mice seroconverted, but BtΔ*cdaA*-infected mice did not (data not shown). This result suggested that BtΔ*cdaA* failed to stimulate a strong humoral immune response because the mutant was cleared early during infection. In all, these murine infection studies support the hypothesis that CdaA is critical for B. turicatae mammalian infection.

### The BtΔ*cdaA* mutational strategy results in polar mutation effects.

**FIG 3:**
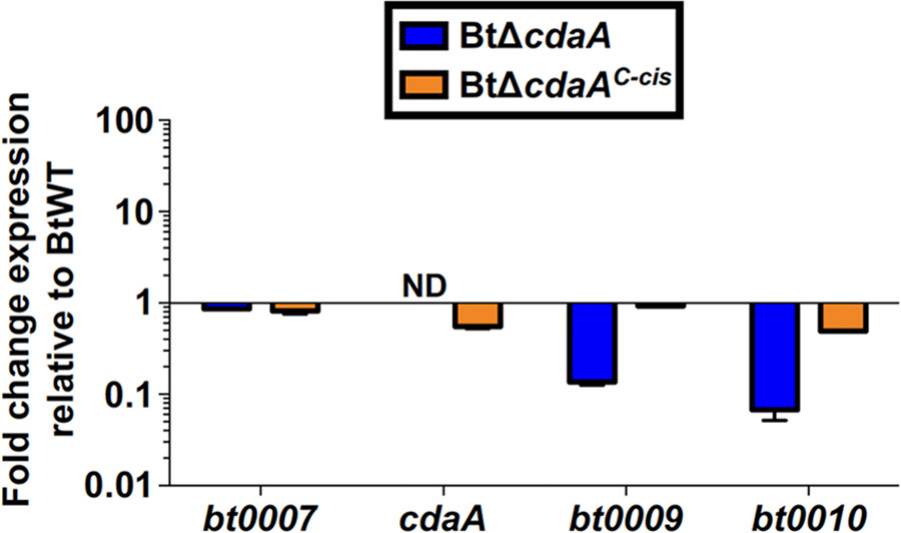
Polar mutation effects associated with the BtΔ*cdaA* mutational strategy. cDNA generated from BtWT, BtΔ*cdaA*, and BtΔ*cdaA^C-cis^* RNA was used for qRT-PCR analyses to measure expression of *bt0007*, *cdaA*, *bt0009*, and *bt0010*. Expression was normalized to *flaB*, and error bars represent SEM. Shown are results from two biological replicates, and fold change relative to BtWT was calculated using the 2^−ΔΔ^*^CT^* method. ND, not detected.

Because the 3′ end of *cdaA* is located only 4 bp upstream of the coding region for *bt0009* and *cdaA* is encoded on the same strand as the six genes that are immediately downstream, it was possible that our BtΔ*cdaA* mutational approach may result in polar mutation effects. Quantitative reverse transcription-PCR (qRT-PCR) was used to measure expression of *cdaA* and adjacent genes in *in vitro*-cultured BtWT, BtΔ*cdaA*, and BtΔ*cdaA^C-cis^* (Fig. 3). As expected, *cdaA* transcript was undetected in BtΔ*cdaA* spirochetes, while expression was restored in the BtΔ*cdaA^C-cis^* strain, albeit at reduced levels relative to BtWT (0.55-fold change in expression). Expression of *bt0007*, the gene immediately upstream of *bt0008*, was unaffected in BtΔ*cdaA*, but expression of the downstream genes *bt0009* and *bt0010* was reduced approximately 10-fold in the BtΔ*cdaA* strain relative to BtWT. Importantly, this polar mutation effect was near-completely reversed upon complementation in the BtΔ*cdaA^C-cis^* strain (mean fold change in expression of 0.93 and 0.50 for *bt0009* and *bt0010*, respectively, relative to BtWT). These results implied that the murine infection defect observed with BtΔ*cdaA* could be due to either mutation of *cdaA* or to polar mutation effects associated with the mutational strategy. Therefore, additional experiments were required to determine if CdaA was essential for mammalian infection.

### Addressing polar mutation effects associated with the BtΔ*cdaA* mutant.

**FIG 4:**
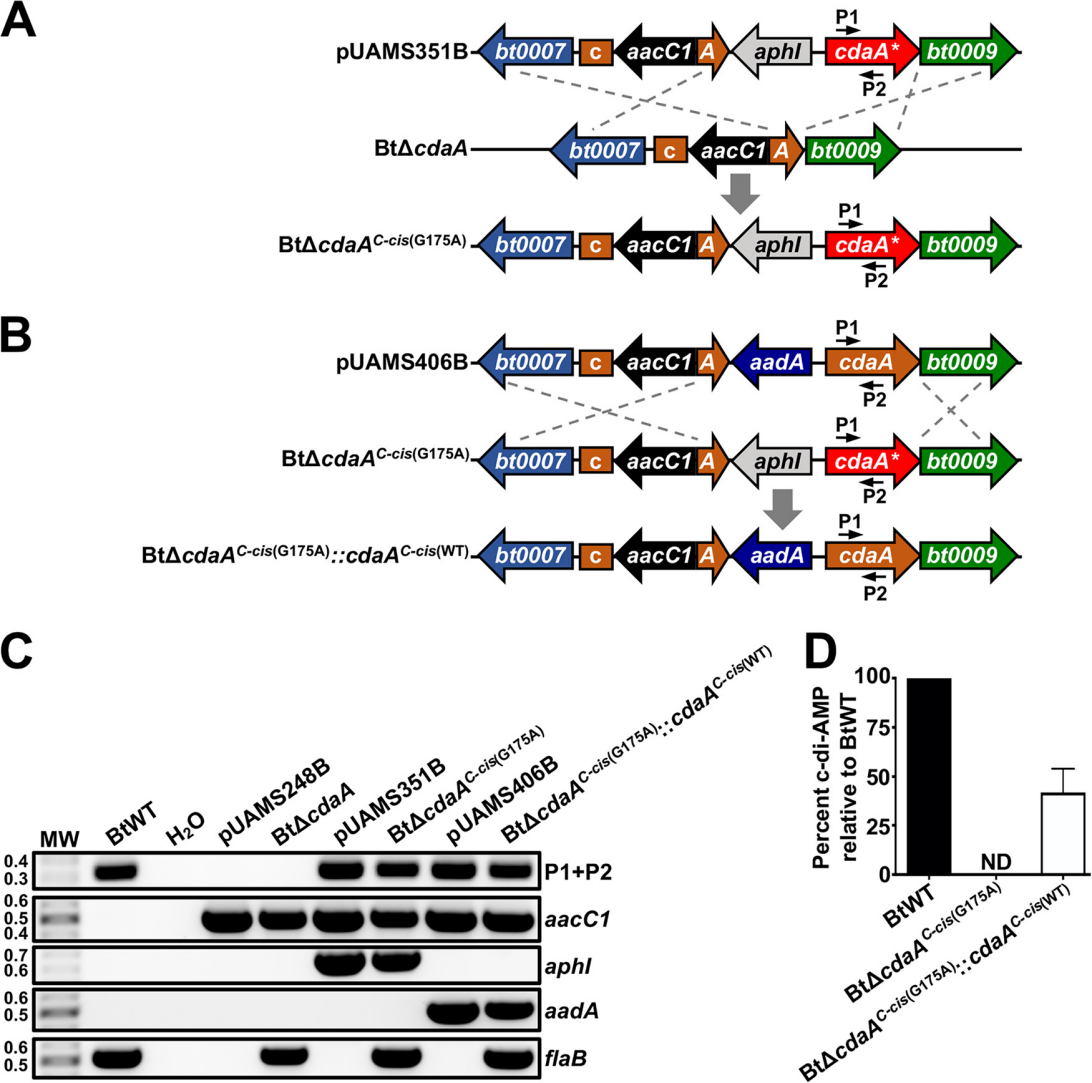
Generation of BtΔ*cdaA^C-cis^*^(G175A)^ and BtΔ*cdaA^C-cis^*^(G175A)^::*cdaA^C-cis^*^(WT)^ strains. (A) Generation of BtΔ*cdaA^C-cis^*^(G175A)^. An *aphI* resistance cassette and a mutated copy of *cdaA* containing a single nucleotide change, which led to a G175A mutation (cdaA*), were inserted into the BtΔ*cdaA* genome adjacent to the site of mutation. Shown are relevant regions of the pUAMS351B complementation construct and BtΔ*cdaA* and BtΔ*cdaA^C-cis^*^(G175A)^ chromosomes. Numbered arrows represent approximate locations of primers used in panel C. (B) Generation of BtΔ*cdaA^C-cis^*^(G175A)^::*cdaA^C-cis^*^(WT)^. The *aphI* resistance cassette and mutated copy of *cdaA* in BtΔ*cdaA^C-cis^*^(G175A)^ were replaced with an *aadA* resistance cassette and a wild-type copy of *cdaA*. Shown are relevant regions of the pUAMS406B complementation construct and BtΔ*cdaA^C-cis^*^(G175A)^ and BtΔ*cdaA^C-cis^*^(G175A)^::*cdaA^C-cis^*^(WT)^ chromosomes. Numbered arrows represent approximate locations of primers used in panel C. (C) Genotypic confirmation of BtΔ*cdaA^C-cis^*^(G175A)^ and BtΔ*cdaA^C-cis^*^(G175A)^::*cdaA^C-cis^*^(WT)^. PCRs were performed with BtWT, BtΔ*cdaA*, BtΔ*cdaA^C-cis^*^(G175A)^, and BtΔ*cdaA^C-cis^*^(G175A)^::*cdaA^C-cis^*^(WT)^ strains to amplify an internal region of *cdaA* (P1+P2; 328 bp), *aacC1* (489 bp), *aphI* (624 bp), *aadA* (463 bp), or *flaB* (519 bp). pUAMS248B, pUAMS351B, and pUAMS406B were used as positive controls for BtΔ*cdaA*, BtΔ*cdaA^C-cis^*^(G175A)^ and BtΔ*cdaA^C-cis^*^(G175A)^::*cdaA^C-cis^*^(WT)^, respectively, and PCR with no template (H_2_O) served as a contamination control. MW denotes the DNA standard, and numbers to the left indicate molecular weight in kb. (D) c-di-AMP quantification in BtWT, BtΔ*cdaA^C-cis^*^(G175A)^, and BtΔ*cdaA^C-cis^*^(G175A)^::*cdaA^C-cis^*^(WT)^. Strains were grown to late exponential phase, and c-di-AMP levels in harvested cells were measured. Percent c-di-AMP in each strain relative to BtWT is graphed, and error bars represent SEM. ND, not detected.

Because the BtΔ*cdaA* mutational strategy resulted in polar mutation effects on genes downstream of *bt0008* in the chromosome, we were unable to directly attribute the mammalian infection defect in BtΔ*cdaA* to inactivation of *cdaA*. As complementation of BtΔ*cdaA* in the BtΔ*cdaA^C-cis^* strain resulted in near-complete reversal of the polar mutational effect, we hypothesized that a similar strategy could be used to introduce a copy of *cdaA* with a point mutation rendering the encoded protein incapable of DAC activity into BtΔ*cdaA*, thereby generating a *cdaA* mutant without the significant polar mutation effects. This mutant could subsequently be genetically complemented by replacing the point mutant *cdaA* allele with a wild-type copy to maintain similar expression of adjacent genes in mutant and complemented strains. Rosenburg et al. previously identified a conserved glycine residue in CdaA homologs that can be mutated to an alanine to abrogate DAC activity (90). Using an approach analogous to that employed for generation of BtΔ*cdaA^C-cis^*, the BtΔ*cdaA* strain was transformed with a construct to insert the *aphI* kanamycin resistance cassette and a point mutant copy of *cdaA*, which contained a single-nucleotide change converting the glycine encoded at position 175 to an alanine (G175A), adjacent to the original mutagenesis site, creating BtΔ*cdaA^C-cis^*^(G175A)^ (Fig. 4A). BtΔ*cdaA^C-cis^*^(G175A)^ was then complemented by replacing the kanamycin resistance marker and G175A-mutated allele of *cdaA* with an *aadA* streptomycin resistance cassette and wild-type copy of *cdaA*, designated BtΔ*cdaA^C-cis^*^(G175A)^::*cdaA^C-cis^*^(WT)^ (Fig. 4B). Consistent with the presence of *cdaA* alleles in BtΔ*cdaA^C-cis^*^(G175A)^ and BtΔ*cdaA^C-cis^*^(G175A)^::*cdaA^C-cis^*^(WT)^, both strains were positive by PCR for *cdaA*, while the BtΔ*cdaA* mutant screened negative (Fig. 4C). As expected, BtΔ*cdaA^C-cis^*^(G175A)^ and BtΔ*cdaA^C-cis^*^(G175A)^::*cdaA^C-cis^*^(WT)^ were positive for the kanamycin and streptomycin resistance markers, respectively, whereas BtΔ*cdaA*, BtΔ*cdaA^C-cis^*^(G175A)^, and BtΔ*cdaA^C-cis^*^(G175A)^::*cdaA^C-cis^*^(WT)^ were all PCR positive for the gentamicin marker. Additionally, PCR for *flaB* resulted in amplicons of the expected size in all B. turicatae strains. We further confirmed the presence and absence of the point mutation in *cdaA* in BtΔ*cdaA^C-cis^*^(G175A)^ and BtΔ*cdaA^C-cis^*^(G175A)^::*cdaA^C-cis^*^(WT)^, respectively, by PCR amplifying and Sanger sequencing an internal region of *cdaA* (data not shown). Finally, high-performance liquid chromatography-tandem mass spectrometry (HPLC-MS/MS) analyses confirmed that BtΔ*cdaA^C-cis^*^(G175A)^ failed to produce c-di-AMP. Alternatively, c-di-AMP was readily detectable in BtΔ*cdaA^C-cis^*^(G175A)^::*cdaA^C-cis^*^(WT)^, but an approximate 2-fold reduction in c-di-AMP levels was observed relative to BtWT (see Discussion) (Fig. 4D).

**FIG 5:**
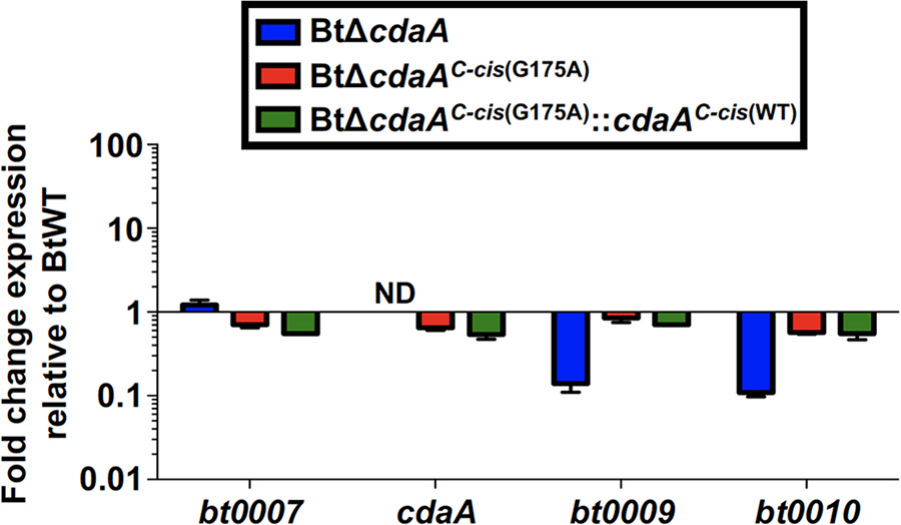
Expression of genes adjacent to *cdaA* in BtΔ*cdaA^C-cis^*^(G175A)^ and BtΔ*cdaA^C-cis^*^(G175A)^::*cdaA^C-cis^*^(WT)^. cDNA generated from BtWT, BtΔ*cdaA*, BtΔ*cdaA^C-cis^*^(G175A)^, and BtΔ*cdaA^C-cis^*^(G175A)^::*cdaA^C-cis^*^(WT)^ RNA was used for qRT-PCR analyses to measure expression of *bt0007*, *cdaA*, *bt0009*, and *bt0010*. Expression was normalized to *flaB*, and error bars represent SEM. Shown are results from two biological replicates, and fold change relative to BtWT was calculated using the 2^−ΔΔ^*^CT^* method. ND, not detected.

qRT-PCR analyses were performed to assess *in vitro* expression of genes adjacent to *cdaA* in BtΔ*cdaA^C-cis^*^(G175A)^ and BtΔ*cdaA^C-cis^*^(G175A)^::*cdaA^C-cis^*^(WT)^ (Fig. 5). As expected, polar mutation effects observed in BtΔ*cdaA* were near-completely reversed in BtΔ*cdaA^C-cis^*^(G175A)^ and BtΔ*cdaA^C-cis^*^(G175A)^::*cdaA^C-cis^*^(WT)^. Although modest reductions in expression were observed in *bt0008*, *bt0009*, and *bt0010* (ranging from 0.54- to 0.85-fold relative to BtWT), levels of *bt0007*, *cdaA*, *bt0009*, and *bt0010* transcripts were similar in BtΔ*cdaA^C-cis^*^(G175A)^ and BtΔ*cdaA^C-cis^*^(G175A)^::*cdaA^C-cis^*^(WT)^. These data imply that any phenotypic differences observed between these strains cannot be attributed to polar mutation effects. Therefore, BtWT, BtΔ*cdaA^C-cis^*^(G175A)^, and BtΔ*cdaA^C-cis^*^(G175A)^::*cdaA^C-cis^*^(WT)^ were used in subsequent experiments to further characterize the impact of the *cdaA* mutation (see below).

### BtΔ*cdaA^C-cis^*^(G175A)^ is significantly attenuated in a murine needle-challenge model of RF.

**FIG 6:**
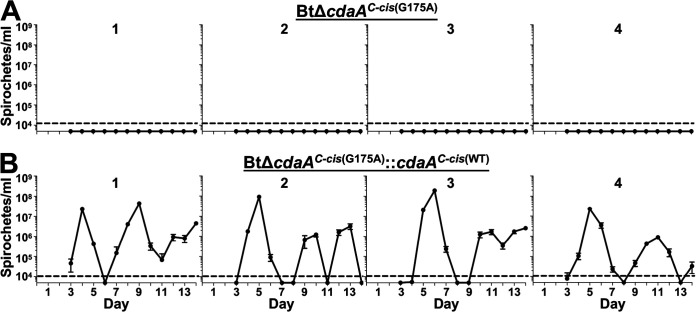
Murine infection phenotypes of BtΔ*cdaA^C-cis^*^(G175A)^ and BtΔ*cdaA^C-cis^*^(G175A)^::*cdaA^C-cis^*^(WT)^ strains. Groups of four mice were intradermally inoculated with 10^2^ spirochetes of BtΔ*cdaA^C-cis^*^(G175A)^ (A) or BtΔ*cdaA^C-cis^*^(G175A)^::*cdaA^C-cis^*^(WT)^ (B). On days 3 to 14 postinfection, bacterial levels in the bloodstream were quantified by qPCR. Numbers above the graphs indicate individual mice in each experimental group, and error bars represent SEM. The dashed line indicates the LOD (10^4^ spirochetes/ml).

To assess if the G175A point mutation in *cdaA* resulted in attenuated mammalian infection, groups of four mice were intradermally inoculated with 10^2^ BtΔ*cdaA^C-cis^*^(G175A)^ or BtΔ*cdaA^C-cis^*^(G175A)^::*cdaA^C-cis^*^(WT)^ bacteria (Fig. 6). While BtΔ*cdaA^C-cis^*^(G175A)^ spirochetes were not detected on any day postinfection in the bloodstream by qPCR (Fig. 6A), BtΔ*cdaA^C-cis^*^(G175A)^::*cdaA^C-cis^*^(WT)^-infected mice reached levels of 10^6^ to 10^8^ bacteria/ml of blood during recurrent peaks (Fig. 6B), which is consistent with BtWT infection (Fig. 2A). Furthermore, bacteria could not be cultured from the blood of BtΔ*cdaA^C-cis^*^(G175A)^-infected mice, but BtΔ*cdaA^C-cis^*^(G175A)^::*cdaA^C-cis^*^(WT)^ spirochetes were cultured from the blood on days postinfection when spirochetemia was detectable by qPCR (data not shown). Finally, all four BtΔ*cdaA^C-cis^*^(G175A)^-infected mice failed to seroconvert when BtWT lysates were probed with serum collected 14 days postinfection, indicating the bacteria were likely eliminated early during infection. Conversely, all four mice infected with the BtΔ*cdaA^C-cis^*^(G175A)^::*cdaA^C-cis^*^(WT)^ strain seroconverted (data not shown). These results indicate that DAC activity of CdaA is crucial for B. turicatae mammalian infection and that polar mutation effects were not the sole reason for the attenuation seen with BtΔ*cdaA* (Fig. 2).

### *cdaA* mutants contain chromosomal single-nucleotide polymorphisms.

Because c-di-AMP signaling is essential for growth in many bacterial systems under standard culture conditions, it was possible that suppressor mutations were required to generate the BtΔ*cdaA* mutant (34, 40, 56, 60–63). To test this, WGS was performed on two independently generated BtΔ*cdaA* clones and the parental BtWT strain. These analyses identified SNPs in both mutants (Table 1). BtΔ*cdaA* clone 1 had two missense mutations in the chromosomal genes *bt0380* and *bt0531*, which encode an MgtE family Mg^2+^ transporter and *N*-acetylmuramoyl-l-alanine amidase, respectively. MgtE family transporters are widespread in eukaryotes and prokaryotes and primarily transport divalent cations, such as Mg^2+^ and Co^2+^ (91, 92). *N*-acetylmuramoyl-l-alanine amidases are ubiquitous bacterial proteins involved in turnover of the cell wall (93). In BtΔ*cdaA* clone 2, four chromosomal SNPs were discovered. Missense mutations were identified in *bt0219* and *bt0747*, which encode a ZIP family metal transporter and an oligopeptide transport system permease protein (OppB), respectively. ZIP family metal transporters are found in eukaryotes and prokaryotes, and they generally have broad specificity for divalent cations, including Zn^2+^, Fe^2+^, and Mn^2+^ (94, 95). OppB serves as a membrane permease that is part of the borrelial oligopeptide transport system (96). Additionally, nonsense mutations were found in *bt0127* and *bt0241B*, which encode the 30S ribosomal protein S1 and a glycerophosphoryl diester phosphodiesterase (GlpQ), respectively. The 30S ribosomal protein S1 is a component of the prokaryotic 30S ribosomal subunit that participates in binding and unfolding of specific mRNA structures and allows for correct ribosomal positioning during translation initiation (97). GlpQ cleaves deacylated phospholipids to glycerol-3-phosphate, which can be used for phospholipid biosynthesis or be shunted into the glycolytic pathway via a dihydroxyacetone phosphate intermediate (98). In all, WGS of BtΔ*cdaA* clones identified SNPs in proteins involved in membrane transport, metabolism, and translation, possibly representing compensatory suppressor mutations required for normal physiology during *in vitro* growth. Of note, BtΔ*cdaA* clone 1 was used for initial infection experiments (Fig. 2), as well as for derivation of BtΔ*cdaA^C-cis^*^(G175A)^ and BtΔ*cdaA^C-cis^*^(G175A)^::*cdaA^C-cis^*^(WT)^ clones used herein.

**TABLE 1:** Single-nucleotide polymorphisms identified in BtΔ*cdaA* mutants

Strain	Gene	Encoded protein^*a*^	Mutation	Result
BtΔ*cdaA* clone 1	*bt0380*	MgtE	Missense (G→T)	K(160)→N
*bt0531*	*N*-acetylmuramoyl-l-alanine amidase	Missense (G→A)	E(133)→K	
BtΔ*cdaA* clone 2	*bt0127*	30S ribosomal protein S1	Nonsense (G→T)	E(401)→Stop
*bt0219*	ZIP family metal transporter	Missense (C→T)	P(104)→S	
*bt0241B*	GlpQ	Nonsense (C→T)	W(235)→Stop	
*bt0747*	OppB	Missense (G→A)	G(186)→R	

a MgtE, Mg^2+^ transporter; GlpQ, glycerophosphoryl diester phosphodiesterase; OppB, oligopeptide transport system permease protein.

### *cdaA* is required for normal growth and physiology *in vitro*.

**FIG 7:**
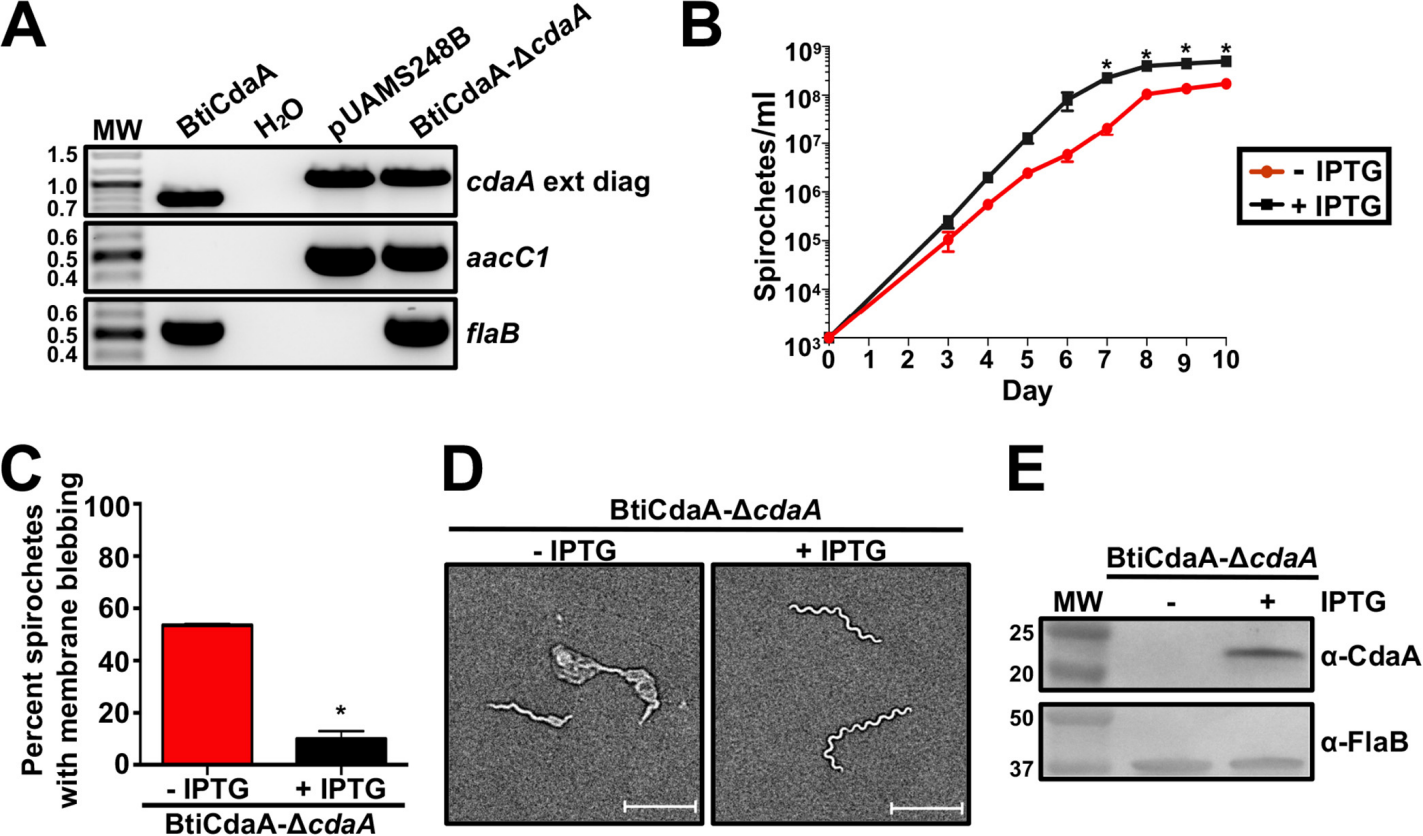
Conditional mutation of *cdaA*. (A) Genotypic confirmation of BtiCdaA-Δ*cdaA.* PCRs were performed with BtiCdaA and BtiCdaA-Δ*cdaA* to amplify a region flanking the site of mutation (*cdaA* ext diag; BtiCdaA, 803 bp, and BtiCdaA-Δ*cdaA*, 1,066 bp), as well as an internal region of *aacC1* (489 bp) and *flaB* (519 bp). pUAMS248B served as a positive control for BtiCdaA-Δ*cdaA*, and a PCR without template (H_2_O) was performed as a purity control. MW denotes the DNA standard, and numbers to the left indicate molecular weight in kb. (B) Impact of CdaA depletion on growth. BtiCdaA-Δ*cdaA* spirochetes were grown to late exponential phase with 1 mM IPTG, washed twice with mBSK media to remove IPTG, and then inoculated at 10^3^ bacteria/ml with (+) or without (−) 1 mM IPTG. Bacteria were then enumerated by dark-field microscopy daily beginning on day 3 postinoculation. Displayed are results from two biological replicates, and error bars represent SEM. *, *P < *0.05. (C) Impact of CdaA depletion on morphology. BtiCdaA-Δ*cdaA* spirochetes were grown with (+) or without (−) 1 mM IPTG to late exponential phase and examined by dark-field microscopy. One hundred spirochetes from two biological replicates were examined for membrane blebbing, and error bars represent SEM. *, *P < *0.05. (D) Imaging of morphological defects associated with depletion of CdaA. Representative bright-field images of BtiCdaA-Δ*cdaA* spirochetes grown with (+) or without (−) 1 mM IPTG from the experiment in panel C are displayed. Two biological replicates were examined, and a representative image from one replicate is shown. The scale bar equals 10 μm. (E) Immunoblot analyses of BtiCdaA-Δ*cdaA* cultured with (+) or without (−) IPTG. Whole-cell lysates were prepared after completion of growth curve experiments in panel B. Proteins were separated by SDS-PAGE and then transferred to a nitrocellulose membrane. Membranes were then probed with antiserum or antibody for CdaA or FlaB, respectively. Antiserum/antibodies used to detect the respective proteins are indicated to the right. MW denotes the protein standard, and numbers to the left indicate molecular weight in kDa. Two biological replicates were performed, yielding similar results, and a representative blot from one replicate is shown.

The identification of SNPs in two independently generated BtΔ*cdaA* clones could imply that suppressor mutations are required for *in vitro* viability in the absence of CdaA. To evaluate this possibility, a conditional mutational strategy was used (99, 100). A B. turicatae shuttle vector carrying a *lacI* cassette and *cdaA* under transcriptional control of a *lac*-inducible promoter was transformed into BtWT, generating BtiCdaA. BtiCdaA was then transformed with the Δ*cdaA* mutational construct and grown in the presence of 1 mM isopropyl-β-d-thiogalactopyranoside (IPTG) to maintain *cdaA* expression until use of the conditional mutant in CdaA depletion experiments. Genotypic confirmation of the conditional mutant, designated BtiCdaA-Δ*cdaA*, was achieved by PCR to amplify a region flanking the site of mutation, as well as a PCR to amplify an internal region of the *aacC1* gene (Fig. 7A). The PCR flanking the site of mutation (*cdaA* ext diag) revealed amplicons of the appropriate sizes in BtiCdaA and BtiCdaA-Δ*cdaA*, while the PCR for *aacC1* only generated a product in the BtiCdaA-Δ*cdaA* strain, consistent with successful mutagenesis. PCRs for *flaB* resulted in amplicons of the appropriate size in BtiCdaA and BtiCdaA-Δ*cdaA* strains.

To determine whether depletion of CdaA resulted in an *in vitro* physiological defect, BtiCdaA-Δ*cdaA* was grown to late exponential phase, washed twice in media to remove IPTG, and then inoculated at an initial density of 10^3^ bacteria/ml of modified Barbour-Stoenner-Kelly (mBSK) medium with or without 1 mM IPTG. Growth was then quantified daily by dark-field microscopy (Fig. 7B). Interestingly, BtiCdaA-Δ*cdaA* grown in the absence of IPTG exhibited a growth defect, with significantly reduced densities on days 7 to 10 postinoculation relative to BtiCdaA-Δ*cdaA* cultured with IPTG. BtiCdaA-Δ*cdaA* cultured without IPTG also exhibited extensive membrane blebbing visible by bright-field microscopy (Fig. 7C and D). In fact, 53.5% of BtiCdaA-Δ*cdaA* spirochetes grown without IPTG had visual membrane blebbing, while only 10% of BtiCdaA-Δ*cdaA* bacteria grown with IPTG had membrane blebs. Finally, to confirm successful depletion of CdaA, immunoblot analyses were performed (Fig. 7E). As expected, CdaA was readily detectable in BtiCdaA-Δ*cdaA* cultured with IPTG, but the protein was undetectable in BtiCdaA-Δ*cdaA* grown without IPTG. Blots for FlaB were comparable regardless of IPTG treatment. Overall, these results indicated that conditional mutation of *cdaA* does not necessarily result in cell death *in vitro*, but CdaA is required for normal bacterial growth and physiology. Importantly, given that the two BtΔ*cdaA* mutants used for WGS did not have an *in vitro* growth defect or any noticeable morphological changes by dark-field microscopy (data not shown), it is likely that the identified SNPs represent suppressor mutations which contribute to the c-di-AMP-responsive physiology of these clones *in vitro*.

### Inactivation of *cdaA* does not impact *bosR* or *rpoS* expression or protein production.

**FIG 8:**
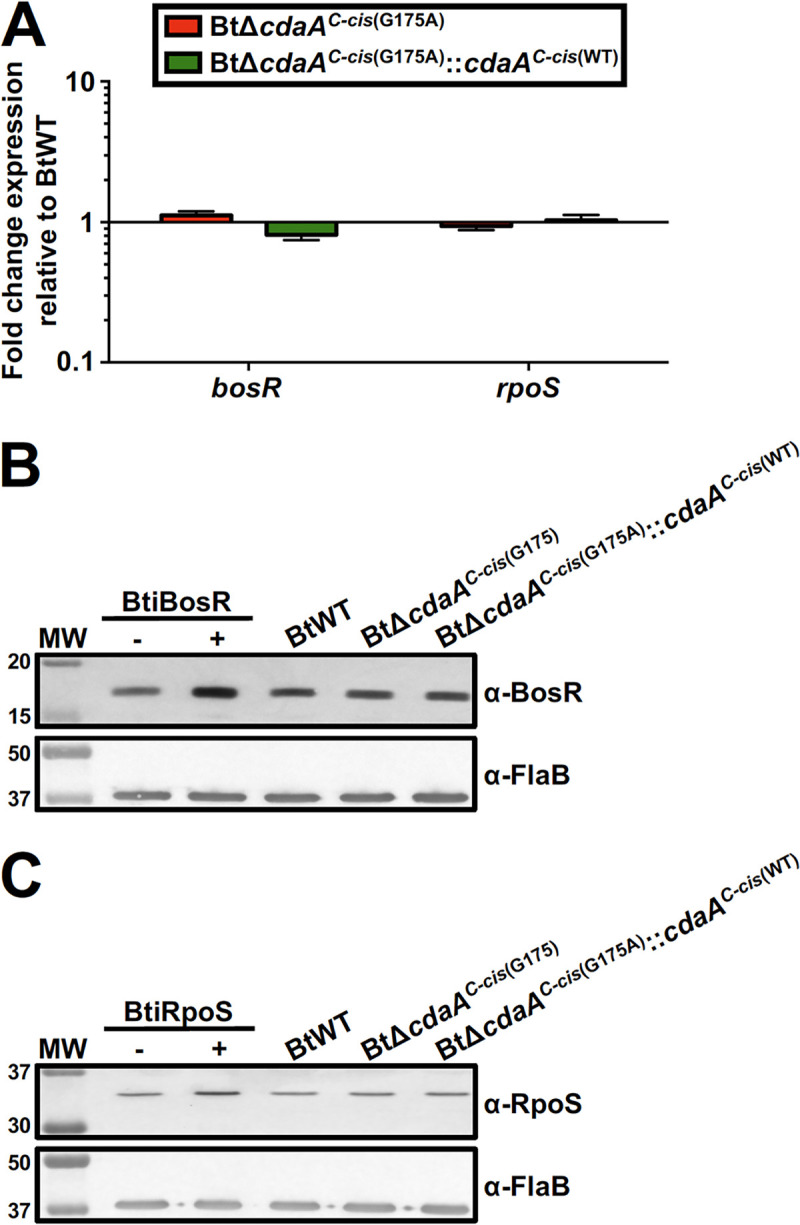
Impact of *cdaA* mutation on *bosR* and *rpoS* expression or protein production. (A) Expression of *bosR* and *rpoS* in the *cdaA* mutant. cDNA generated from BtWT, BtΔ*cdaA^C-cis^*^(G175A)^, and BtΔ*cdaA^C-cis^*^(G175A)^::*cdaA^C-cis^*^(WT)^ RNA was used for qRT-PCR analyses to measure expression of *bosR* and *rpoS*. Expression was normalized to *flaB*, and error bars represent SEM. Shown are results from four biological replicates, and fold change relative to BtWT was calculated using the 2^−ΔΔ^*^CT^* method. (B) Effect of *cdaA* mutation on BosR production. BtiBosR was grown to mid-exponential phase and treated with 1 mM IPTG (+) or left untreated (0 mM IPTG) (−) for 48 h, and BtWT, BtΔ*cdaA^C-cis^*^(G175A)^, and BtΔ*cdaA^C-cis^*^(G175A)^::*cdaA^C-cis^*^(WT)^ were grown to late exponential phase. Whole-cell lysates were prepared, and proteins were separated by SDS-PAGE, transferred to a nitrocellulose membrane, and probed with antiserum or antibody against BosR or FlaB, respectively. Antiserum/antibodies used to detect the respective proteins are indicated to the right. MW denotes the protein standard, and numbers to the left indicate molecular weight in kDa. Four biological replicates were performed, yielding similar results, and a representative blot from one replicate is shown. (C) Effect of *cdaA* mutation on RpoS production. BtiRpoS was grown to mid-exponential phase and treated with 1 mM IPTG (+) or left untreated (0 mM IPTG) (−) for 24 h, and BtWT, BtΔ*cdaA^C-cis^*^(G175A)^, and BtΔ*cdaA^C-cis^*^(G175A)^::*cdaA^C-cis^*^(WT)^ strains were grown to late exponential phase. Whole-cell lysates were prepared, and proteins were separated by SDS-PAGE, transferred to a nitrocellulose membrane, and probed with antiserum or antibody against RpoS or FlaB, respectively. Antiserum/antibodies used to detect the respective proteins are indicated to the right. MW denotes the protein standard, and numbers to the left indicate molecular weight in kDa. Four biological replicates were performed, yielding similar results, and a representative blot from one replicate is shown.

In B. burgdorferi, the RpoS alternative sigma factor controls expression of several known virulence determinants and is essential for virulence (101–103). *Borrelia* oxidative stress regulator (BosR) in B. burgdorferi binds upstream of *rpoS* and serves as a transcriptional activator (104–107). Not surprisingly, BosR is also required for mammalian infection by B. burgdorferi (104, 106). Ye et al. demonstrated that conditional depletion of the c-di-AMP PDE, DhhP, in B. burgdorferi resulted in increased levels of c-di-AMP, as well as modestly reduced expression of *bosR* and significantly reduced expression of *rpoS* (64). BosR and RpoS protein levels were also markedly reduced, resulting in decreased production of virulence factors (64). This led us to hypothesize that mutation of *cdaA* could impact BosR and/or RpoS production in B. turicatae, possibly contributing to the observed infection defect. To evaluate this hypothesis, *bosR* and *rpoS* transcription (Fig. 8A) and protein production (Fig. 8B and C) were measured in *in vitro* cultured BtWT, BtΔ*cdaA^C-cis^*^(G175A)^, and BtΔ*cdaA^C-cis^*^(G175A)^::*cdaA^C-cis^*^(WT)^. Interestingly, there were only modest changes in expression of either regulator in BtΔ*cdaA^C-cis^*^(G175A)^ or BtΔ*cdaA^C-cis^*^(G175A)^::*cdaA^C-cis^*^(WT)^ relative to BtWT (1.12- and 0.81-fold change, respectively, for *bosR*; 0.94- and 1.03-fold change, respectively, for *rpoS*). Immunoblot analyses also revealed no substantial changes in BosR or RpoS production between BtWT, BtΔ*cdaA^C-cis^*^(G175A)^, and BtΔ*cdaA^C-cis^*^(G175A)^::*cdaA^C-cis^*^(WT)^, and levels of FlaB, which served as a loading control, were equivalent in the three strains. To confirm the specificity of the BosR and RpoS antiserum used in immunoblot analyses, a B. turicatae shuttle vector carrying a *lacI* cassette and either *bosR* or *rpoS* under transcriptional control of a *lac*-inducible promoter was transformed into BtWT, generating the strains BtiBosR and BtiRpoS, respectively. As expected, when BtiBosR and BtiRpoS were treated with 1 mM IPTG, increases in BosR and RpoS protein levels, respectively, were observed. In all, mutation of *cdaA* appeared to have no effect on *bosR* and *rpoS* transcription or protein production *in vitro*. This observation strongly suggests that c-di-AMP-mediated gene regulation differs between RF and LD spirochetes.

### The *cdaA* mutant is sensitive to increased salt.

**FIG 9:**
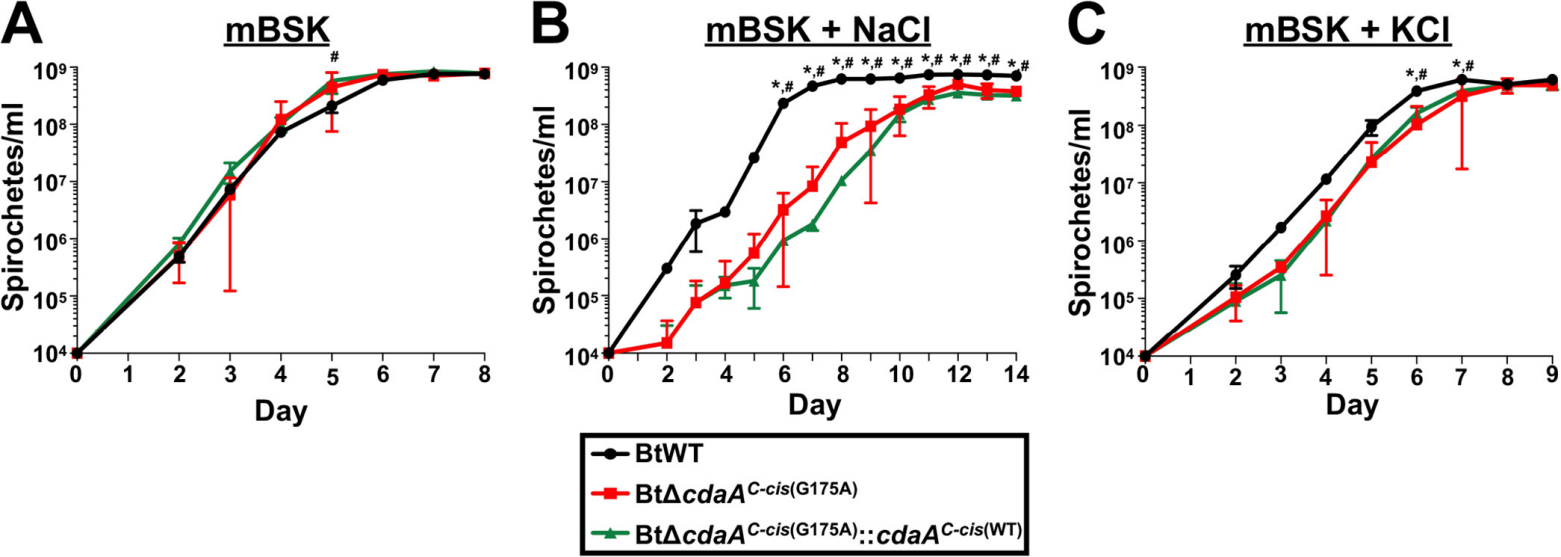
Impact of NaCl and KCl on growth of BtΔ*cdaA^C-cis^*^(G175A)^ and BtΔ*cdaA^C-cis^*^(G175A)^::*cdaA^C-cis^*^(WT)^. BtWT, BtΔ*cdaA^C-cis^*^(G175A)^, and BtΔ*cdaA^C-cis^*^(G175A)^::*cdaA^C-cis^*^(WT)^ cultures were inoculated at a density of 10^4^ spirochetes/ml in mBSK medium (A), mBSK supplemented with 50 mM NaCl (B), or mBSK supplemented with 50 mM KCl (C). Bacterial densities were then quantified by dark-field microscopy daily beginning on day 2 postinoculation. Displayed are results from two biological replicates, and error bars represent SEM. *, *P < *0.05, BtΔ*cdaA^C-cis^*^(G175A)^ relative to BtWT; #, *P < *0.05, BtΔ*cdaA^C-cis^*^(G175A)^::*cdaA^C-cis^*^(WT)^ relative to BtWT.

Considering the critical role of c-di-AMP signaling in osmoregulation in many bacterial systems, it is not surprising that mutation of c-di-AMP signaling pathway components results in altered susceptibility to salt treatment (42–44, 50, 58, 65, 108). To determine if mutation of *cdaA* in B. turicatae also results in altered resistance to salt treatment, the maximum concentrations of NaCl and KCl at which BtWT could grow was first determined using previously described MIC plating assays (109, 110). BtWT was able to grow in concentrations up to 50 mM for each salt (data not shown), so these concentrations were used in subsequent growth curve analyses with BtWT, BtΔ*cdaA^C-cis^*^(G175A)^, and BtΔ*cdaA^C-cis^*^(G175A)^::*cdaA^C-cis^*^(WT)^ strains (Fig. 9). In standard mBSK media, BtΔ*cdaA^C-cis^*^(G175A)^ and BtΔ*cdaA^C-cis^*^(G175A)^::*cdaA^C-cis^*^(WT)^ strains showed modestly increased growth rates relative to BtWT, with BtΔ*cdaA^C-cis^*^(G175A)^::*cdaA^C-cis^*^(WT)^ having a significantly increased density on day 5 postinoculation (see Discussion) (Fig. 9A). With 50 mM NaCl supplementation, all strains grew slower than that seen in mBSK media (Fig. 9B). However, BtΔ*cdaA^C-cis^*^(G175A)^ had a growth defect relative to BtWT, exhibiting significantly lower numbers of bacteria on 6 to 14 days postinoculation, and genetic complementation of BtΔ*cdaA^C-cis^*^(G175A)^ failed to reverse this growth phenotype. Significant growth defects were also observed in BtΔ*cdaA^C-cis^*^(G175A)^ and BtΔ*cdaA^C-cis^*^(G175A)^::*cdaA^C-cis^*^(WT)^ relative to BtWT when grown with 50 mM KCl; both strains had significantly reduced numbers of bacteria on days 6 to 7 postinoculation (Fig. 9C). The inability to complement the salt-dependent growth defect in BtΔ*cdaA^C-cis^*^(G175A)^ suggests that (i) suppressor mutations, (ii) decreased c-di-AMP levels, or (iii) modestly reduced expression of genes downstream of *cdaA* in BtΔ*cdaA^C-cis^*^(G175A)^ and BtΔ*cdaA^C-cis^*^(G175A)^::*cdaA^C-cis^*^(WT)^ strains could be responsible for the phenotype. Therefore, more evidence is required to elucidate a possible role for CdaA in survival under conditions of increased osmolarity (see Discussion).

### Mutation of *cdaA* has no effect on growth under nutrient-limited conditions but results in a significant growth defect at decreased osmolarity.

**FIG 10:**
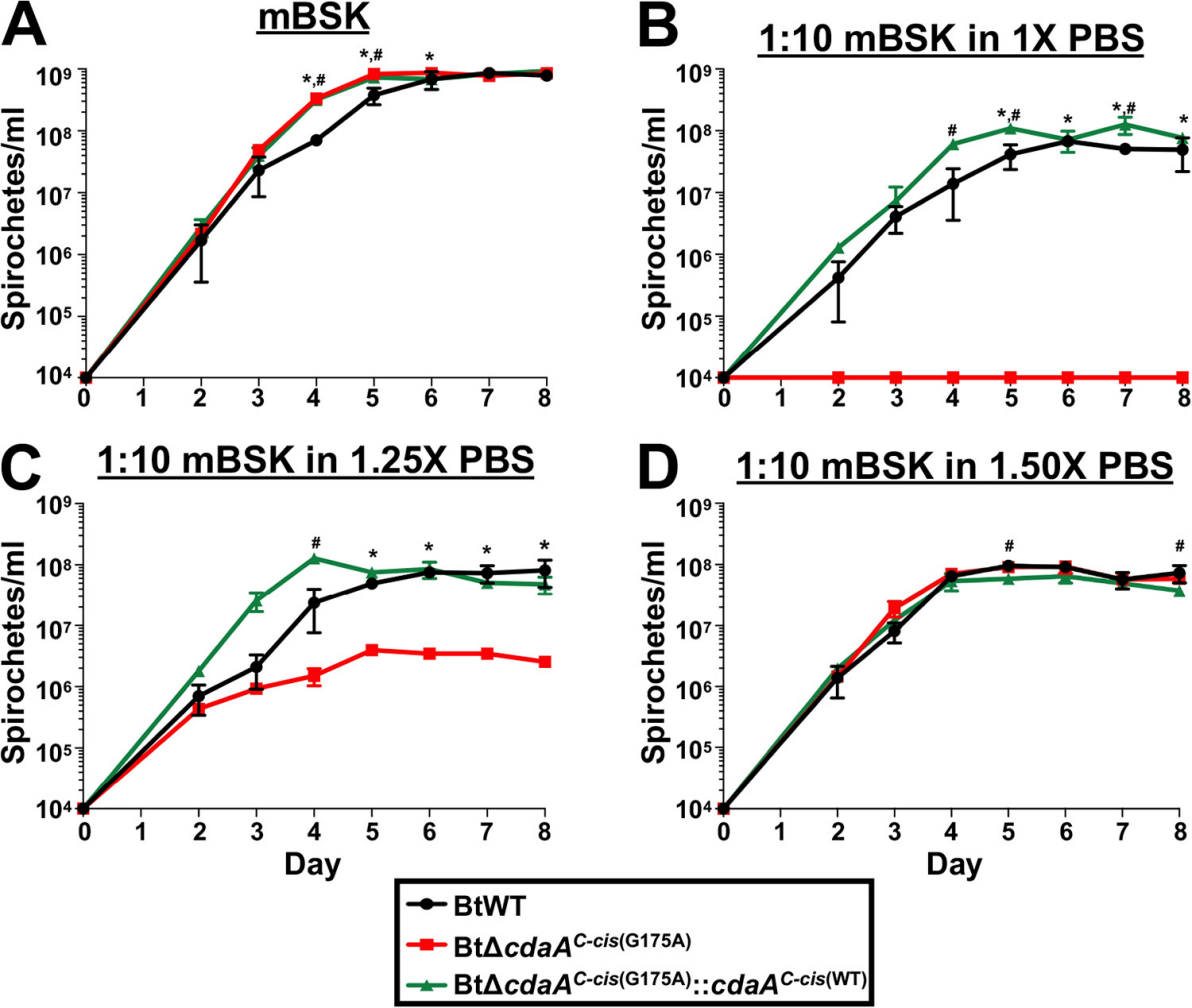
Impact of reduced osmolarity on BtΔ*cdaA^C-cis^*^(G175A)^ growth. BtWT, BtΔ*cdaA^C-cis^*^(G175A)^, and BtΔ*cdaA^C-cis^*^(G175A)^::*cdaA^C-cis^*^(WT)^ cultures were inoculated at a density of 10^4^ spirochetes/ml in mBSK medium (A) or mBSK diluted 1:10 in 1× PBS (B), 1.25× PBS (C), or 1.50× PBS (D). Bacteria were then enumerated by dark-field microscopy daily beginning on day 2 postinoculation. Displayed are results from two biological replicates, and error bars represent SEM. *, *P < *0.05, BtΔ*cdaA^C-cis^*^(G175A)^ relative to BtWT; #, *P < *0.05, BtΔ*cdaA^C-cis^*^(G175A)^::*cdaA^C-cis^*^(WT)^ relative to BtWT.

Because c-di-AMP signaling and the bacterial stress response have been linked in S. aureus and L. monocytogenes (55–57), it was possible that CdaA may play a role in survival under nutrient-limited conditions. To assess this hypothesis, growth curve analyses were performed with BtWT, BtΔ*cdaA^C-cis^*^(G175A)^, and BtΔ*cdaA^C-cis^*^(G175A)^::*cdaA^C-cis^*^(WT)^ strains in either standard mBSK media (Fig. 10A) or in media diluted 1:10 in 1× phosphate-buffered saline (PBS) to simulate nutrient-limited conditions (Fig. 10B) (111, 112). As expected, BtΔ*cdaA^C-cis^*^(G175A)^ and BtΔ*cdaA^C-cis^*^(G175A)^::*cdaA^C-cis^*^(WT)^ strains exhibited an increased growth rate in standard mBSK relative to the BtWT strain (see Fig. 9A). In media diluted 1:10 in 1× PBS, however, BtΔ*cdaA^C-cis^*^(G175A)^ failed to reach numbers detectable by dark-field microscopy. Alternatively, BtWT and BtΔ*cdaA^C-cis^*^(G175A)^::*cdaA^C-cis^*^(WT)^ strains were able to grow in the dilute media, indicating that CdaA is required for growth under these conditions.

It should be noted that the osmolarity of mBSK medium is 450 mOsm, and the osmolarity of 1× PBS is 300 mOsm (137 mM NaCl, 2.7 mM KCl, and 11.9 mM phosphates) (113). Therefore, dilution of the media with PBS alters both the nutrient composition and osmolarity of the media. Given the role of c-di-AMP signaling in osmoregulation in other bacteria, a possibility for the observed growth defect in media diluted 1:10 in 1× PBS could be the decrease in osmolarity (58). To address this alternative, growth curves were performed with BtWT, BtΔ*cdaA^C-cis^*^(G175A)^, and BtΔ*cdaA^C-cis^*^(G175A)^::*cdaA^C-cis^*^(WT)^ in mBSK diluted 1:10 in 1.25× PBS (375 mOsm 171.25 mM NaCl, 3.38 mM KCl, and 14.88 mM phosphates) (Fig. 10C) and mBSK diluted 1:10 in 1.50× PBS (450 mOsm 205.5 mM NaCl, 4.05 mM KCl, and 17.85 mM phosphates) (Fig. 10D). BtWT and BtΔ*cdaA^C-cis^*^(G175A)^::*cdaA^C-cis^*^(WT)^ grew similarly in mBSK diluted 1:10 in 1.25× PBS, but BtΔ*cdaA^C-cis^*^(G175A)^ exhibited a growth defect with significantly reduced densities on days 5 to 8 postinoculation. BtΔ*cdaA^C-cis^*^(G175A)^ was not able to grow in mBSK diluted in 1× PBS, but detectable bacterial densities of BtΔ*cdaA^C-cis^*^(G175A)^ (e.g., maximum of 10^6^ bacteria/ml) were observed in mBSK diluted in 1.25× PBS. Interestingly, in mBSK diluted 1:10 in 1.50× PBS, which is the same osmolarity as mBSK, growth was similar between all three strains. These data imply that, rather than being required to tolerate nutrient-limited conditions *in vitro*, CdaA is essential for growth at decreased osmolarity, suggesting a critical role for c-di-AMP in osmoregulation in B. turicatae.

### The *cdaA* mutant requires pyruvate for growth *in vitro*.

**FIG 11:**
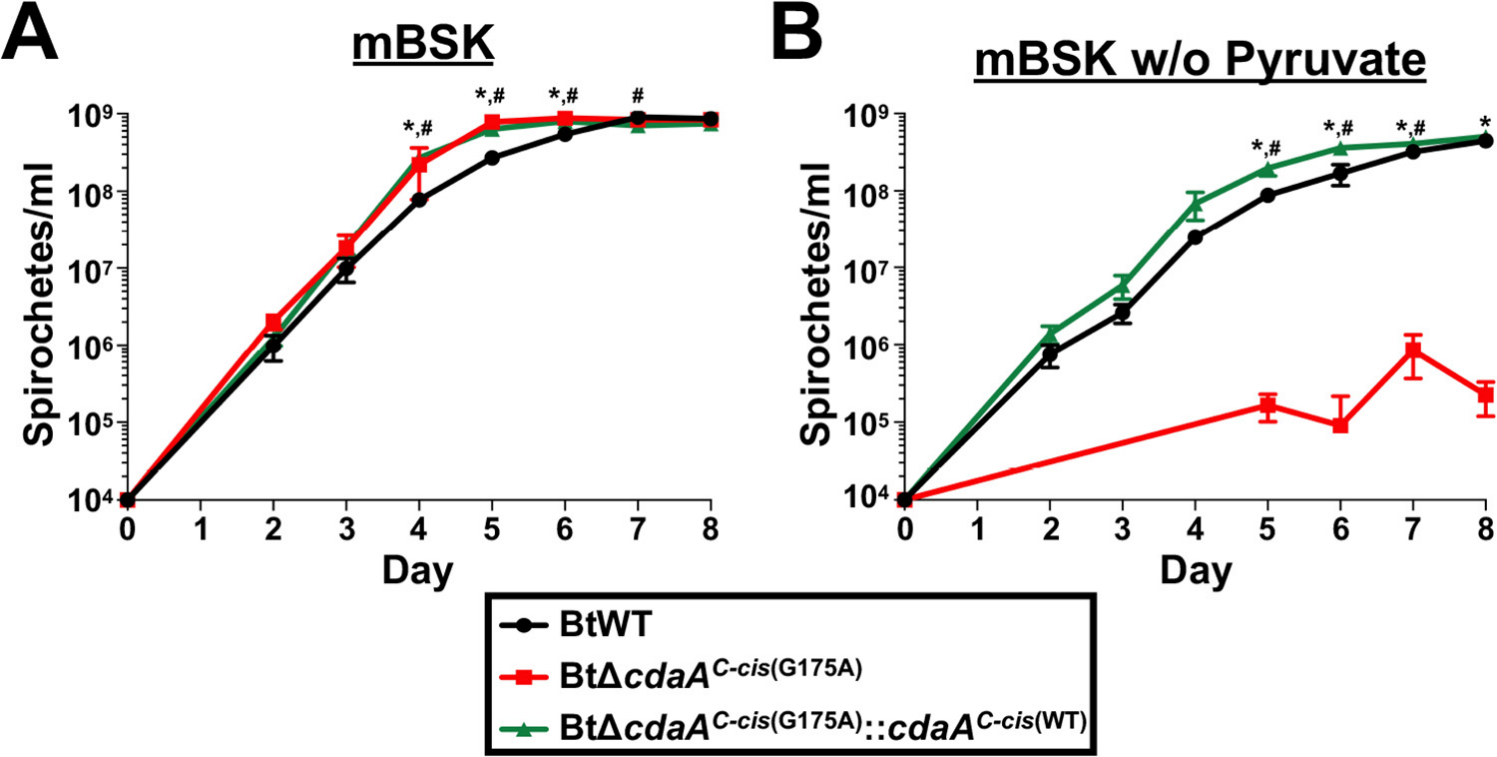
Assessing the requirement of pyruvate for BtΔ*cdaA^C-cis^*^(G175A)^ growth. BtWT, BtΔ*cdaA^C-cis^*^(G175A)^, and BtΔ*cdaA^C-cis^*^(G175A)^::*cdaA^C-cis^*^(WT)^ cultures were inoculated at a density of 10^4^ spirochetes/ml in mBSK medium (A) or mBSK lacking pyruvate (B). Bacterial densities were then quantified by dark-field microscopy daily beginning on day 2 postinoculation. Displayed are results from two biological replicates, and error bars represent SEM. *, *P < *0.05, BtΔ*cdaA^C-cis^*^(G175A)^ relative to BtWT; #, *P < *0.05, BtΔ*cdaA^C-cis^*^(G175A)^::*cdaA^C-cis^*^(WT)^ relative to BtWT.

c-di-AMP signaling is important for resistance to ROS in other bacterial systems (41, 51–53). Therefore, experiments were performed to assess if mutation of *cdaA* in B. turicatae led to altered susceptibility to oxidative stresses. Experiments to measure the sensitivity of *Borrelia* spirochetes to ROS are typically performed using media lacking pyruvate, as pyruvate can act as an ROS scavenger and decrease the assay sensitivity (89, 114, 115). When attempting to test the susceptibility of the *cdaA* mutant to oxidative agents, it was serendipitously found that BtΔ*cdaA^C-cis^*^(G175A)^ failed to reach an adequate density in mBSK medium lacking pyruvate. Following this observation, growth curve analyses were performed to quantify differences in growth of BtWT, BtΔ*cdaA^C-cis^*^(G175A)^, and BtΔ*cdaA^C-cis^*^(G175A)^::*cdaA^C-cis^*^(WT)^ in mBSK with or without pyruvate (Fig. 11). As expected, in the normal media formulation, BtΔ*cdaA^C-cis^*^(G175A)^ and BtΔ*cdaA^C-cis^*^(G175A)^::*cdaA^C-cis^*^(WT)^ had modestly increased growth rates relative to the BtWT strain (Fig. 11A). However, BtΔ*cdaA^C-cis^*^(G175A)^ exhibited a growth defect in mBSK lacking pyruvate, reaching significantly lower bacterial densities than BtWT on days 5 to 8 postinoculation (Fig. 11B). Importantly, genetic complementation in BtΔ*cdaA^C-cis^*^(G175A)^::*cdaA^C-cis^*^(WT)^ reversed this phenotype. Therefore, while the contribution of *cdaA* to ROS resistance could not be determined using media lacking pyruvate (see Discussion), pyruvate was found to be critical for growth of the *cdaA* mutant *in vitro*. Given the critical role of pyruvate in bacterial metabolism, these results possibly link c-di-AMP signaling to an important function in central metabolism in B. turicatae.

## DISCUSSION

RF is a significant global public health concern, but no literature exists regarding regulatory pathways required for virulence of the causative *Borrelia* spirochetes (4, 13, 14, 116, 117). Given the essential role of c-di-AMP signaling in bacterial virulence, we sought to assess the function of this dinucleotide second messenger in the TBRF spirochete B. turicatae by inactivating the sole DAC, CdaA (44, 45, 54, 64, 67–71). In a murine needle-challenge infection model, *cdaA* mutants were unable to establish bloodstream infection like BtWT (Fig. 2 and 6). Furthermore, mice infected with *cdaA* mutants failed to seroconvert, indicating that the bacteria were likely eliminated soon after inoculation and before mice could develop a strong B-cell response (118–121). Importantly, this infection defect was reversed by genetic complementation, indicating that putative suppressor mutations important for *in vitro* growth in the absence of CdaA (Table 1) and modestly decreased expression of genes downstream of *cdaA* in BtΔ*cdaA^C-cis^*^(G175A)^ (Fig. 5) were not responsible for the defect. Of note, it is possible that the infection defect exhibited by *cdaA* mutants could be overcome by increasing the dose, as only 10^2^ spirochetes were used for the murine model described herein. However, Boyle et al. demonstrated that only 1 to 10 spirochetes can be transmitted during feeding by Ornithodoros turicata, the vector for B. turicatae, during natural infection (122). We use an inoculum of 10^2^ spirochetes because this was the closest dose to natural infection that we could accurately deliver while achieving a 100% infection rate with consistent bacteremic relapses in BtWT-infected mice. Therefore, our dose was approximately 10- to 100-fold higher than could be possibly transmitted by the tick vector, and increasing the inoculum would be further inconsistent with respect to the enzootic cycle of B. turicatae. It is worth noting that infection experiments performed with the BtΔ*cdaA* mutant at a dose of 10^4^ spirochetes (1,000- to 10,000-fold higher than numbers transmitted during natural infection) demonstrated that this strain was still unable to infect mice like BtWT (data not shown). This suggests that the infection defect observed in *cdaA* mutants could likely not be overcome by increasing the inoculum. Ultimately, our data indicate that CdaA is critical for infection in a biologically relevant needle-inoculation model of infection, but it is still necessary to assess the role of CdaA in transmission from tick to mammal using the experimental murine-tick infection model. Intriguingly, c-di-AMP signaling pathways are absent in many Gram-negative organisms, but they are conserved in the *Spirochaetes* phylum (78). This conservation, as well as the results reported herein and in B. burgdorferi, may suggest a possible role for the signaling pathway in the virulence of other pathogenic spirochetes, such as *Leptospira* and *Treponema* species (64).

The initial BtΔ*cdaA* mutational strategy, which replaced an internal region of the *cdaA* ORF with a gentamicin resistance cassette, resulted in approximately 10-fold reduced expression of the downstream genes *bt0009* and *bt0010*. The fact that this polar mutation was almost completely reversed upon genetic complementation (Fig. 3 and 5) could indicate that a promoter region controlling expression of these downstream genes is located within the *cdaA* ORF. However, in *Firmicutes*, *cdaA* is the first gene of a conserved three-gene operon. *cdaA* is typically followed by genes encoding the cyclic di-AMP synthase A regulator (CdaR) and the phosphoglucosamine mutase GlmM (48, 65, 123–126). In these bacteria, the N-terminal domains anchor both CdaA and CdaR to the membrane, and the C-terminal domains of CdaA and CdaR are located intracellularly and extracellularly, respectively. CdaR interacts with the transmembrane domain of CdaA to regulate DAC activity (48, 65, 123–125). GlmM, an intracellular enzyme involved in cell wall biosynthesis, also directly interacts with CdaA to modulate DAC activity (124–126). In B. turicatae, the gene immediately downstream of *cdaA*, *bt0009*, has been annotated as a YbbR domain-containing protein (a domain found in CdaR proteins of *Firmicutes*), possibly indicating that BT0009 could function as a CdaR homolog (48, 65, 123–126). However, *bt0010* does not encode a GlmM homolog; rather, the gene encodes a holo-(acyl-carrier-protein) synthase, an enzyme involved in fatty acid metabolism (127). RT-PCR analyses revealed that *cdaA*, *bt0009*, and *bt0010* are transcriptionally linked (data not shown). Additionally, the four genes downstream of *bt0010* are encoded on the same strand with no more than 4 bp separating the ORFs, suggesting these genes could also be part of the *cdaA*-containing operon. These genes encode two hypothetical proteins, tRNA pseudouridine synthase A and primosomal protein N′. Future studies will aim to define the *cdaA*-containing operon and examine transcriptional regulation at this locus. Additionally, future work will assess if BT0009, BT0010, and other possible proteins encoded in the operon have a function related to c-di-AMP signaling.

In this study, we found that the genomes of two independently generated *cdaA* mutant clones contained SNPs, which likely represent suppressor mutations that compensate and allow normal growth and physiology in the absence of CdaA *in vitro* (Table 1 and Fig. 7). The presence of suppressor mutations upon *dac* mutation is consistent with reports in other bacteria (54, 56, 61, 128–132). BtΔ*cdaA* clone 1 had SNPs in genes encoding an MgtE family transporter and an *N*-acetylmuramoyl-l-alanine amidase. Interestingly, an MgtE homolog in Bacillus subtilis binds c-di-AMP via the conserved cystathionine beta-synthase (CBS) domain (130). This could suggest that the identified missense mutation enables appropriate MgtE-mediated regulation of Mg^2+^ or Co^2+^ homeostasis *in vitro* when the protein is unable to bind c-di-AMP. To our knowledge, *N*-acetylmuramoyl-l-alanine amidase proteins have not been specifically linked to c-di-AMP signaling. However, due to the extensive role of c-di-AMP in cell wall metabolism in other bacteria, the identification of this SNP is not surprising (133). Of note, BtΔ*cdaA* clone 1 was used for generation of BtΔ*cdaA^C-cis^*^(G175A)^, which was subsequently complemented to make BtΔ*cdaA^C-cis^*^(G175A)^::*cdaA^C-cis^*^(WT)^. All three of these strains exhibit a significant increase in growth rates relative to BtWT, while BtΔ*cdaA* clone 2 exhibits no difference in growth (Fig. 9A, Fig. 10A, and Fig. 11A; data not shown). This observation suggests that a SNP or combination of SNPs in BtΔ*cdaA* clone 1 may be responsible for the increased growth rate. Interestingly, mutation of *mgtE* in the bacterium Shewanella oneidensis results in an increased growth rate in the presence of certain divalent metals (134). Future examination of the growth of BtΔ*cdaA* clone 1 in different media formulations, as well as generation and phenotypic analysis of a B. turicatae mutant containing the point mutation in *mgtE*, will address the possibility that this SNP is responsible for the altered growth kinetics.

BtΔ*cdaA* clone 2 had SNPs in genes encoding the 30S ribosomal protein S1, a ZIP family metal transporter, a glycerophosphoryl diester phosphodiesterase (GlpQ), and an oligopeptide transport system permease protein (OppB). Interestingly, suppressor mutations in genes encoding subunits of Opp oligopeptide transport systems have also been noted upon *dac* mutation in L. monocytogenes and S. agalactiae (54, 56, 129). Given that oligopeptides can serve as osmolytes, this conserved relationship between c-di-AMP signaling and oligopeptide transport could be related to bacterial osmoregulation (54, 135). Alternatively, this relationship could suggest roles for c-di-AMP in amino acid metabolism. Further studies are required to examine these possibilities. To our knowledge, suppressor mutations in genes encoding ZIP family transporters, glycerophosphoryl diester phosphodiesterase proteins, or ribosomal proteins have not been reported upon *dac* mutation in other bacteria. As MgtE and ZIP family transporters both transport diverse divalent cations, the mutation in the gene encoding the ZIP family protein in BtΔ*cdaA* clone 2 could serve an analogous compensatory function to the mutation in *mgtE* in BtΔ*cdaA* clone 1 in the absence of CdaA (91, 92, 94, 95). The potential suppressor mutation in *glpQ* may support a role for c-di-AMP in lipid metabolism or glycolysis, and the growth defect in the *cdaA* mutant in the absence of pyruvate (Fig. 11) may further implicate c-di-AMP in control of the glycolytic pathway (discussed below) (98). Finally, the nonsense mutation in the gene encoding the 30S ribosomal protein S1 may suggest a role for c-di-AMP in translation initiation. Of note, all attempts to transform BtΔ*cdaA* clone 2 with pUAMS351B to generate a second Δ*cdaA^C-cis^*^(G175A)^ clone failed to result in insertion of the *aphI* resistance marker and mutated *cdaA* allele. However, kanamycin-resistant bacteria were readily isolated. Subsequent MIC plating assays revealed that BtΔ*cdaA* clone 2 was hyperresistant to kanamycin, with an MIC >300 μg/ml (data not shown). This observation implicates one or more of these suppressor mutations in resistance to kanamycin. Interestingly, mutation of Opp family proteins has been implicated in increased resistance to aminoglycoside antibiotics, as Opps can facilitate antibiotic permeation of the Gram-negative inner membrane (136–138). Further investigation is required to determine if the SNP in *oppB* results in kanamycin hyperresistance. In all, WGS of BtΔ*cdaA* clones identified SNPs in genes encoding proteins involved in membrane transport, metabolism, and translation. Additional studies are required to elucidate whether these individual mutations in BtΔ*cdaA* clones 1 and 2 can reverse the physiological and growth defects seen upon depletion of CdaA (Fig. 7). Future work will also seek to identify if c-di-AMP regulates expression or activity of these genes or gene products, respectively.

During complementation, we confirmed that the BtΔ*cdaA^C-cis^*^(G175A)^ mutant lacked c-di-AMP, but approximately 2-fold less c-di-AMP was measured in the BtΔ*cdaA^C-cis^*^(G175A)^::*cdaA^C-cis^*^(WT)^-complemented strain than BtWT (Fig. 4D). This observation could be due to the modest reduction in *cdaA* expression in BtΔ*cdaA^C-cis^*^(G175A)^::*cdaA^C-cis^*^(WT)^ relative to BtWT (mean, 0.54-fold change) (Fig. 5), which correlated with the measured fold difference in c-di-AMP production. However, Savage et al. reported that significantly altering production of CdaA in B. burgdorferi had little effect on c-di-AMP levels, indicating that DAC activity is tightly controlled through an unknown mechanism (78). It is likely, given the critical role of c-di-AMP in B. turicatae, that RF spirochetes also tightly regulate c-di-AMP production. This observation leads to two alternative possibilities for decreased c-di-AMP levels in BtΔ*cdaA^C-cis^*^(G175A)^::*cdaA^C-cis^*^(WT)^. First, modestly reduced expression of *bt0009* is observed in the complemented strain relative to BtWT (0.71-fold change). Heterologous co-overexpression of the *cdaA* and *cdaR* homologs of B. subtilis in Escherichia coli results in significantly increased c-di-AMP levels relative to expression of *cdaA* alone, indicating that CdaR could serve as a positive regulator of CdaA DAC activity (123). Therefore, it is logical that reduced *bt0009* expression in the complemented strain may lead to decreased c-di-AMP levels, assuming that BT0009 acts as a CdaR homolog. A second, and more intriguing, explanation for reduced c-di-AMP levels in BtΔ*cdaA^C-cis^*^(G175A)^::*cdaA^C-cis^*^(WT)^ could be due to the acquisition of additional suppressor mutations. As mutations are required for normal physiology and growth in the absence of CdaA (Fig. 7), it is possible that restoration of CdaA in a mutant adapted for growth in the absence of c-di-AMP could also lead to detrimental effects. It is therefore possible that additional suppressor mutations were required for generation of the complemented mutant. These suppressor mutations could lie within genes encoding proteins which regulate CdaA activity either directly or indirectly, thus leading to altered DAC activity. Although outside the scope of the current manuscript, future studies will evaluate the possibility of additional suppressor mutations in BtΔ*cdaA^C-cis^*^(G175A)^::*cdaA^C-cis^*^(WT)^. Importantly though, despite the inability to fully restore c-di-AMP levels in BtΔ*cdaA^C-cis^*^(G175A)^::*cdaA^C-cis^*^(WT)^, all phenotypes associated with *cdaA* mutation, aside from the growth defect upon treatment with salts, were reversed using our complementation approach.

Although c-di-AMP regulates global gene expression in B. burgdorferi through control of *bosR* and *rpoS* expression, mutation of *cdaA* had no discernible effect on expression or protein production of these regulatory orthologs in B. turicatae (Fig. 8) (64). In B. burgdorferi, expression of numerous virulence-associated genes is controlled via the RpoN-RpoS alternative sigma factor pathway (101–103, 139, 140). The pathway(s) regulating virulence gene expression in RF spirochetes remains to be determined, but B. turicatae and other New World RF spirochetes have a point mutation in the *rpoN* gene, which significantly truncates the protein (141). Therefore, RF spirochetes may have evolved a divergent signaling pathway to control expression of virulence genes, and it is possible that c-di-AMP impacts this pathway. It should be noted, however, that, while depletion of DhhP in B. burgdorferi led to changes in the proteome visible by SDS-PAGE and Coomassie brilliant blue (CBB) staining, no changes were visible via these analyses upon mutation of *cdaA* in B. turicatae (data not shown) (64). While this observation could imply that c-di-AMP does not impact global expression in B. turicatae, it is also possible that c-di-AMP impacts expression of genes which encode proteins not produced at high enough levels to be detected by CBB staining. Therefore, future studies will include transcriptomic and proteomic approaches to measure the impact of *cdaA* mutation in B. turicatae on global gene expression and protein production, respectively.

The most well-characterized function of c-di-AMP in bacteria is osmoregulation (58). Herein, we found two lines of evidence supporting a role for c-di-AMP in osmoregulation in B. turicatae. First, the *cdaA* mutant had a significant growth defect when cultured in the presence of NaCl and KCl (Fig. 9). Interestingly, genetic complementation failed to reverse this phenotype, which implicates the identified SNPs, reduced levels of c-di-AMP, and/or modestly decreased expression of genes downstream of *cdaA* in BtΔ*cdaA^C-cis^*^(G175A)^ and BtΔ*cdaA^C-cis^*^(G175A)^::*cdaA^C-cis^*^(WT)^ as possible contributors for the growth defect. While these caveats do not allow us to directly link CdaA to survival during salt treatment, the possibility that a suppressor mutation required for normal growth in the absence of CdaA impacts survival during salt treatment could indirectly link c-di-AMP signaling to osmoregulation. Interestingly, BtΔ*cdaA* clone 1 (as well as strains derived from this clone) and BtΔ*cdaA* clone 2 harbor completely different SNPs, but they exhibited similar growth defects with NaCl and KCl treatment (data not shown). These data could imply that the unique combinations of suppressor mutations in these clones may result in similar, but each with narrower ranges of osmolarity resistance, with the clones being the most well adapted to the osmolarity of mBSK media, 450 mOsm (113). More evidence is required, however, to address this possibility. The second line of evidence supporting a role for c-di-AMP in osmoregulation in B. turicatae was the growth phenotype of the *cdaA* mutant at decreased osmolarity (Fig. 10). The mutant was unable to grow at an osmolarity of 300 mOsm, and, importantly, genetic complementation reversed this growth defect. Growth at 375 mOsm partially rescued this phenotype, and no growth defect was noted at 450 mOsm (the osmolarity of mBSK). Of note, the osmolarity of mammalian blood is 300 mOsm (113). Given that the *cdaA* mutant is unable to grow at 300 mOsm *in vitro* or establish bloodstream infection (Fig. 6), it is tempting to speculate that the infection phenotype is due to this defect in osmoregulation. However, given that c-di-AMP plays roles in other virulence-associated bacterial phenotypes, defects in osmoregulation could represent just one of several reasons why the *cdaA* mutant is significantly attenuated during murine infection (44, 46, 64, 69, 71–76).

In this study, we serendipitously discovered that CdaA is required for normal growth of B. turicatae in mBSK medium lacking pyruvate (Fig. 11), which suggests a role for c-di-AMP signaling in central carbon metabolism, consistent with reports in other bacteria (41, 54, 142, 143). However, these previous studies have revealed only one direct role for c-di-AMP in regulation of central metabolism, which is inhibition of pyruvate carboxylase activity (142, 143). Interestingly, *Borrelia* spirochetes do not encode a pyruvate carboxylase homolog or any protein involved in the tricarboxylic acid (TCA) cycle (77, 144, 145). In fact, the only known fate of pyruvate in *Borrelia* following glycolysis is conversion to lactate by the lactate dehydrogenase enzyme (144). This reaction is one of only three in *Borrelia*, along with the reduction of the disulfide form of coenzyme A (CoA) by CoA disulfide reductase and the nicotinamidase reaction in the nicotinamide salvage pathway that is capable of regenerating NAD^+^ from NADH (144, 146, 147). Importantly, glycolysis has an absolute requirement for the cofactor NAD^+^ (148). Therefore, the growth defect observed in media lacking pyruvate could imply that the *cdaA* mutant is unable to generate sufficient NAD^+^ levels for continued glycolysis and that, by increasing pyruvate levels, the NAD^+^/NADH ratio can be restored to a value necessary for survival. Future studies are required to elucidate the exact role of c-di-AMP in central carbon metabolism in B. turicatae.

c-di-AMP-dependent signaling is important for resistance to oxidative stresses in other bacterial systems (41, 51–53). While we were unable to assess the role of CdaA in resistance to oxidative stresses using media lacking pyruvate, no significant growth defect was observed in the *cdaA* mutant in complete mBSK medium supplemented with oxidizing agents using MIC plating assays or growth curve analyses (data not shown) (89, 109, 110, 115). Another phenotype associated with deletion of c-di-AMP signaling pathway components in other bacteria is altered resistance to β-lactam antibiotics, presumably due to altered cell wall metabolism (38, 40–50). However, using MIC plating assays, we failed to detect any significant changes in susceptibility of the *cdaA* mutant to this class of antimicrobials (data not shown). Finally, c-di-AMP has been linked to survival during heat shock and in low pH, but no defect was observed when growing the *cdaA* mutant in either of these conditions (data not shown) (37–39, 43). The absence of several phenotypes commonly associated with altered c-di-AMP levels in other bacteria is not entirely unexpected since several of these works utilized Gram-positive bacteria (primarily *Firmicutes*). As such, it is important to examine this pathway in an evolutionarily divergent Gram-negative bacterium, as these studies could reveal key functional differences.

In summary, this work represents the first characterization of the DAC, CdaA, in the context of any *Borrelia* spirochete. Importantly, we found that *cdaA* mutants were significantly attenuated in a murine needle-challenge model of RF. Hypothesizing that CdaA could be important for *in vitro* viability, WGS was performed on two mutant clones, which identified SNPs in genes involved in membrane transport, metabolism, and translation, which represent potential suppressor mutations. We also found that depletion of CdaA resulted in a significant growth defect and extensive membrane blebbing. Although c-di-AMP signaling impacts global regulation through control of expression of the virulence regulators *bosR* and *rpoS* in B. burgdorferi, mutation of *cdaA* in B. turicatae had little effect on their respective transcript or protein levels (64). This observation implied that c-di-AMP may impact gene regulation differently in RF and LD spirochetes. Finally, *in vitro* characterization of the *cdaA* mutant revealed significant growth defects with salt treatment, at decreased osmolarity, and in media lacking pyruvate. While the salt treatment phenotype was not reversed after genetic complementation, growth defects at decreased osmolarity and in media lacking pyruvate could be directly attributed to the *cdaA* mutation. These latter observations imply a role for c-di-AMP signaling in osmoregulation and central metabolism in B. turicatae. Future studies will aim to elucidate the mechanism(s) through which c-di-AMP impacts B. turicatae virulence, osmoregulation, and central metabolism by examining the effects of c-di-AMP on global gene regulation, identifying effectors of c-di-AMP, and determining the effect of c-di-AMP binding on these individual effectors. Furthermore, future studies will elucidate how the c-di-AMP signaling pathway is regulated. Lastly, future work will determine if c-di-AMP plays a role in the vector phase of the enzootic cycle.

## MATERIALS AND METHODS

### Bacterial strains and culture conditions.

Bacterial strains used in this study are listed in Table 2. E. coli strain TOP10F′ (Life Technologies, Carlsbad, CA) was used for cloning and plasmid propagation, and E. coli strain C41(DE3) (Lucigen, Middleton, WI) was used for expression of recombinant protein. E. coli was cultured at 37°C in Luria-Bertani (LB) medium supplemented with 100 μg/ml ampicillin, 5 μg/ml gentamicin, 50 μg/ml kanamycin, 100 μg/ml spectinomycin, or 30 μg/ml chloramphenicol when appropriate. Low-passage B. turicatae strain 91E135 (Oz1), designated BtWT, was used in this study (149, 150). B. turicatae strains were passaged no more than twice beyond the original frozen stock. B. turicatae was cultured at 35°C with 3% CO_2_ in modified Barbour-Stoenner-Kelly (mBSK) medium with 12% rabbit serum at pH 7.6 unless noted otherwise (151, 152). mBSK was supplemented with 40 μg/ml gentamicin, 150 μg/ml kanamycin, or 150 μg/ml streptomycin when appropriate.

### Generation of constructs used in this study.

Plasmids and primers used in this study are detailed in Tables 2 and 3, respectively. PrimeStar Max DNA polymerase (TaKaRa Bio, Mountain View, CA) was used for PCR, and all amplicons were TA cloned into pGEM-T Easy (Promega Corp., Fitchburg, WI) and confirmed by Sanger sequencing. For expression of recombinant protein, the full *bosR* ORF (primers 5′ BtBosR ORF BamHI and 3′ BtBosR ORF SpeI), amino acids 120 to 131 of the *rpoS* ORF (primers 5′ BtRpoS-trun_BamHI and 3′ BtRpoS-trun_SpeI), and the *cdaA* ORF lacking the N-terminal region encoding the predicted signal sequence and transmembrane domains (amino acids 1 to 90; primers 5′ *bt0008* w/o TM BamHI and 3′ *bt0008* ORF BamHI) were amplified from genomic BtWT DNA (gDNA). The putative transmembrane domains of CdaA were predicted using TMHMM-2.0 (85, 86). *cdaA*, *rpoS*, an*d bosR* fragments were excised with their respective restriction enzymes and ligated into linearized pProEX-HTb (Life Technologies) to generate pUAMS338, pUAMS233, and pUAMS159, respectively.

**TABLE 2:** Plasmids and strains used in this study

Plasmid or strain	Description^*a*^	Source or reference no.
Plasmids		
pGEM-T Easy	TA cloning vector; Amp^r^	Promega
pProEX-HTb	Expression plasmid; N-terminal, TEV-cleavable His_6_ tag; Amp^r^	Invitrogen
pUAMS159	pProEX-HTb::*bosR*; Amp^r^	This study
pUAMS338	pProEX-HTb::*cdaA* (without the coding region for the putative transmembrane domains); Amp^r^	This study
pUAMS233	pProEX-HTb::*rpoS*^(120-131)^; Amp^r^	This study
pUAMS4	pGEM-T Easy::P*flgB*-*aacC1* (AscI flanked); Gent^r^, Amp^r^	89
pUAMS248B	Δ*cdaA* mutagenesis construct; Gent^r^, Amp^r^	This study
pUAMS309	pGEM-T Easy::P*flaB*-*aphI* (BamHI flanked); Kan^r^, Amp^r^	This study
pUAMS313B	Δ*cdaA^C-cis^* complementation construct; Kan^r^, Amp^r^	This study
pUAMS351B	Δ*cdaA^C-cis^*^(G175A)^ complementation construct; Kan^r^, Amp^r^	This study
pUAMS402	pGEM-T Easy::P*flaB*-*aadA* (BamHI flanked); Spec/Strep^r^, Amp^r^	This study
pUAMS406B	Δ*cdaA^C-cis^*^(G175A)^::*cdaA^C-cis^*^(WT)^ complementation construct; Spec/Strep^r^, Amp^r^	This study
pBSV2	B. burgdorferi cp9-based shuttle vector; Kan^r^	153
pJD7	Derivative of pKFSS1 B. burgdorferi shuttle vector; Spec/Strep^r^	154
pJD44	pJD7-based shuttle vector with *aph*[3′]-*IIIa*; Kan^r^	165
pBtSV-JB	B. turicatae shuttle vector; Kan^r^	This study
pJSB104	B. burgdorferi *lac*-inducible expression construct; Spec/Strep^r^	154
pUAMS267A	piRpoS, *lac*-inducible construct for BtWT *rpoS*; Kan^r^	This study
pUAMS346	piCdaA, *lac*-inducible construct for BtWT *bt0008*; Kan^r^	This study
pUAMS446B	piBosR, *lac*-inducible construct for BtWT *bosR*; Kan^r^	This study
pRARE	Plasmid from Rosetta(DE3) cells; encodes rare tRNA codons in E. coli; Cam^r^	Novagen
Strains		
E. coli		
TOP10F’	F′ [*lacI*q Tn*10* (Tet^r^)] *mcrA* Δ(*mrr-hsdRMS-mcrBC*) ϕ80*lacZ*ΔM15 *nupG* Δ*lacX74 recA1 ara*Δ*139* Δ(*ara-leu*)*7697 galU galK rpsL* (Strep^r^) *endA1*	Life Technologies
C41(DE3)	F^−^ *ompT hsdSB* (r_B_^−^ m_B_^−^) *gal dcm* (DE3)	Lucigen
RosettaBlue(DE3)	*endA1 hsdR17* (r_K12_^−^ m_K12_^+^) *supE44 thi-1 recA1 gyrA96 relA1 lac* (DE3) F′[*proA^+^ B^+^ lacIqZ*ΔM15::Tn*10*] pRARE; Cam^r^	Novagen
B. turicatae		
BtWT	B. turicatae *s*train 91E135 (Oz1), tick isolate	149, 150
BtΔ*cdaA*	*cdaA* null mutant, BtWT transformed with pUAMS248B; Gent^r^	This study
BtΔ*cdaA^C-cis^*	*cdaA* null mutant complemented with a wild-type copy of *cdaA* (pUAMS313B); Gent^r^, Kan^r^	This study
BtΔ*cdaA^C-cis^*^(G175A)^	*cdaA* null mutant complemented with *cdaA* containing the G175A point mutation (pUAMS351B); Gent^r^, Kan^r^	This study
BtΔ*cdaA^C-cis^*^(G175A)^::*cdaA^C-cis^*^(WT)^	Δ*cdaA^C-cis^*^(G175A)^ complemented with a wild-type *cdaA* allele (pUAMS406B); Gent^r^, Strep^r^	This study
BtiCdaA	BtWT transformed with pUAMS346; Kan^r^	This study
BtiBosR	BtWT transformed with pUAMS446B; Kan^r^	This study
BtiRpoS	BtWT transformed with pUAMS267A; Kan^r^	This study
BtiCdaA-Δ*cdaA*	Conditional B. turicatae *cdaA* mutant; BtiCdaA transformed with pUAMS248B; Gent^r^, Kan^r^	This study

a Amp, ampicillin; Gent, gentamicin; Kan, kanamycin; Spec, spectinomycin; Strep, streptomycin; Cam, chloramphenicol.

**TABLE 3:** Primers and probe used in this study

Primer designation	Sequence^*a*^^,^^*b*^	Purpose
5′ BtBosR ORF BamHI	**GGATCC**ATGAACAACAATACAATAGAGGTA	Recombinant protein
3′ BtBosR ORF SpeI	**ACTAGT**TAATTATTTTCATAATCAATATTAGATTTTTCTT	Recombinant protein
5′ *bt0008* w/o TM BamHI	**GGATCC**AAAATAATTATGCAAATTGGAAATTTTAATTTATC	Recombinant protein
3′ *bt0008* ORF BamHI	GTTATAAT**GGATCC**TCATTCTATTAATGCAAGATTT	Recombinant protein
5′ BtRpoS-trun_BamHI	**GGATCC**AGAAAAGAAAATTTAATACTCC	Recombinant protein
3′ BtRpoS-trun_SpeI	**ACTAGT**TAATCACTGTTGTCTAAGTTATAT	Recombinant protein
5′ F1 *bt0008* KO	GTTCCCTTACTATAATTAGCTTCGGC	Mutagenesis/cloning
3′ F1 *bt0008* KO_AscI	**GGCGCGCC**TAAAATGCTGATTAAACTTACATCTAATAC	Mutagenesis/cloning
5′ F2 *bt0008* KO_AscI	**GGCGCGCC**GAAACACCTTTACATGATGGAGCAGTC	Mutagenesis/cloning
3′ F2 *bt0008* KO_BssHII	**GCGCGC**TTGGAGAAATTACTTCCAGATGTTCTG	Mutagenesis/cloning
5′ Bt*flaB*-*aphI*-BamHI	**GGATCC**TTGCCGGCAATTCCTAATCAGA	Mutagenesis/cloning
3′ Kan ORF int -NdeI v.2	GGAAATGACTTATGAGCCATATTCAACGGG	Mutagenesis/cloning
5′ Kan ORF int -NdeI v.2	CCCGTTGAATATGGCTCATAAGTCATTTCC	Mutagenesis/cloning
3′ Bt*flaB*-*aphI*-BamHI	**GGATCC**AGTGTTACAACCAATTAACC	Mutagenesis/cloning
5′ Bt*flgB*-BamHI	**GGATCC**AGCACCCGGTAGCAAGTTAAAAAAATTTG	Mutagenesis/cloning
5′ F2 *bt0008* Comp-BamHI	**GGATCC**CATAAACAAAAAATAAATTTAAATTAACAT	Mutagenesis/cloning
3′ F2 *bt0008* Comp-BglII	**AGATCT**GGAGAAATTACTTCCAGATGTTCTG	Mutagenesis/cloning
5′ DAC/*cis bt0008*	GCAGTTTCATTTGATGCTCGATGAG	Mutagenesis/cloning
3′ *bt0008* G175A	CTAATTATGACTGCTGCATCATGTAAAGGTG	Mutagenesis/cloning
5′ *bt0008* G175A	CACCTTTACATGATGCAGCAGTCATAATTAG	Mutagenesis/cloning
3′ DAC/*cis bt0008*	CGAATTCACTAGTGATTGGATCTGGAG	Mutagenesis/cloning
5′ BtP*flaB*_BamHI	**GGATCC**TAATCAGAAAAATGTGGTTGAAGATTATAAA	Mutagenesis/cloning
3′ P*flaB* NdeI	**CATATG**TCATTTCCTCCGTGATAA	Mutagenesis/cloning
5′ Bt*flaB*-*aadA* junc_NdeI	GGAAATGA**CATATG**AGGGAAGCGG	Mutagenesis/cloning
3′ *aadA* ORF_BamHI	**GGATCC**TAGTTTATTTGCCGACTACCTTGGTGAT	Mutagenesis/cloning
5′ UAMS-88 Kan BglII	**AGATCT**GCCCTTGCCGGCAATTCCTAATCAGA	Mutagenesis/cloning
3′ UAMS-88 Kan AscI	**GGCGCGCC**TAGTTTAGAAAAACTCATCGAGCATC	Mutagenesis/cloning
5′ pJD44 ColE1 *ori*	GACTA**GGCGCGCC**GCTAGCCAATGACCAAAATC	Mutagenesis/cloning
3′ pJD44 ColE1 *ori*	CTTGCCGG**AGATCT**AGTGCAGGAAAGAACATG	Mutagenesis/cloning
5′ UAMS BtOri BamHI	**GGATCC**TAAAATCTTCTTGCCCGCATCTTTTAAAAT	Mutagenesis/cloning
3′ UAMS BtOri AscI	**GGCGCGCC**ATTATCCCCACCTCAGGGTTTCTTT	Mutagenesis/cloning
5′ Bt-P*flgB*/BamHI-HindIII	**GGATCC**TAG**AAGCTT**AGCACCCGGTAGCAAGTTAAA	Mutagenesis/cloning
3′ Bt-P*flgB*/BbLacI junc	GTAACAGGTTTCATATAACCCTCTATATCAC	Mutagenesis/cloning
5′ Bt-P*flgB*/BbLacI junc	GATATAGAGGGTTATATGAAACCTGTTACTTTG	Mutagenesis/cloning
3′ Bt-BbLacI/BglII	**AGATCT**TTATTACTGGCCGCTTTCTAGCCTGG	Mutagenesis/cloning
5′ BtRpoS ORF-NdeI	**CATATG**AATATATTTAGCAATGAAGATTTAAAC	Mutagenesis/cloning
3′ BtRpoS ORF-HindIII	**AAGCTT**ATTGATAAAGTTCTTCTTTGAGTTTTTTTAG	Mutagenesis/cloning
5′ *bt0008* ORF NdeI	GAATATATTGAATA**CATATG**ATTATGATAGATATAAG	Mutagenesis/cloning
3′ *bt0008* ORF HindIII	**AAGCTT**CATTCTATTAATGCAAGATTTAATTTTTTTTTG ATTTCATTTAAACTTAAATT	Mutagenesis/cloning
5′ BtBosR ORF NdeI	**CATATG**AACAACAATACAATAGAGGTAC	Mutagenesis/cloning
3′ BtBosR ORF HindIII	**AAGCTT**ATTAATTATTTTCATAATCAATATTAG	Mutagenesis/cloning
5′ *bt0008* int diag	CATCATTACATCCATTGGTATCGTGTC	PCR Screening
3′ *bt0008* int diag	AGATACAATGGAATCTAATCTTATGCC	PCR Screening
5′ *bt0008* ext diag	GTGTCATCAAAATCAAATATCAAAGC	PCR Screening
3′ *bt0008* ext diag	GCTCTGTGTCTTGTTCCAAAGGTTTT	PCR Screening
5′ *aacC1* diag	GCAACGATGTTACGCAGCAG	PCR Screening
3′ *aacC1* diag	GCATCACTTCTTCCCGTATGC	PCR Screening
5′ *aphI* diag	CGCGATAATGTCGGGCAATCAGG	PCR Screening
3′ *aphI* diag	ACCGAGGCAGTTCCATAGGATGG	PCR Screening
5′ *aadA* diag	GTGATCGCCGAAGTATCGACTC	PCR Screening
3′ *aadA* diag	CAGGAACCGGATCAAAGAGTTCC	PCR Screening
5′ BtFlaB	CTGGAATGGGTGTTGCAGGA	PCR Screening
3′ BtFlaB	CTCCCTCTTGTTGTGCACCT	PCR Screening
Bt*flaB* F	CCAGCATCATTAGCTGGATCAC	qPCR
Bt*flaB* R	GTTGTGCACCTTCCTGAGC	qPCR
Bt*flaB*-Probe	/5YakYel/TGCAGGTGA/ZEN/AGGTGCGCAGGTT/3IABkFQ/	qPCR
S-*bt0007*_IDT-SYBR	TACTAAGGTTTGGGCTTGTGAA	qRT-PCR
AS-*bt0007*_IDT-SYBR	GTATGATCTACGAAATAACATACGCTAC	qRT-PCR
S-*bt0008*_IDT-SYBR_V2	ACATCCATTGGTATCGTGTCTT	qRT-PCR
AS-*bt0008*_IDT-SYBR_V2	GCAATTGGCAATATGTTTGCTATG	qRT-PCR
S-*bt0009*_IDT-SYBR	TTTAGACCTTGACCGAATAACCT	qRT-PCR
AS-*bt0009*_IDT-SYBR	ATCTTTATTGGCAGTTCATATTCCC	qRT-PCR
2_*bt0010*_IDT-SYBR FWD	ACGAAATCAATAGGATGTGA	qRT-PCR
2_*bt0010*_IDT-SYBR REV	CTTACCAGCTAGACTTTCTAA	qRT-PCR
*bt0647* SYBR/IDT FWD Set 1	GTCGGAATTACTAATGACCCTATCT	qRT-PCR
*bt0647* SYBR/IDT REV Set 1	TTTGGATTTGAGGCAATGTGTAG	qRT-PCR
Bt*rpoS* SYBR FWD	CCGTAAGAGAACACAGACTGATAA	qRT-PCR
Bt*rpoS* SYBR REV	GGCTTTCAGGTCTCCTAGTTT	qRT-PCR
5′ Bt*flaB* SYBR/ABI	AAAAACAGCTGAAGAGCTTGGAAT	qRT-PCR
3′ Bt*flaB* SYBR/ABI	CACCCACATGTACTCTTAATGTCCAT	qRT-PCR
5′ BT0380 SNP Diag	TAGATTCCTCCCCAAAAAGG	SNP Confirmation
3′ BT0380 SNP Diag	AGTGGCAGTTAAGGTAGAAG	SNP Confirmation
5′ BT0531 SNP Diag	GAATCCTTACCTTGAAGCAG	SNP Confirmation
3′ BT0531 SNP Diag	CTCTCAACTCTTCCTAGTGC	SNP Confirmation
5′ BT0127 SNP Diag	GGGCTGTAGTTGAACTTG	SNP Confirmation
3′ BT0127 SNP Diag	CTGGACTCTTTTGTATCTCC	SNP Confirmation
5′ BT0219 SNP Diag	TTGCTGCACCTTCTGGCATA	SNP Confirmation
3′ BT0219 SNP Diag	AATGGCGGAAGAGCTTGGTT	SNP Confirmation
5′ BT0241B SNP Diag	TCCCAACCGTTTTCCCTTAAAC	SNP Confirmation
3′ BT0241B SNP Diag	AAGGTCCAATTCCATCGGCA	SNP Confirmation
5′ BT0747 SNP Diag	GCTGTTGCGAGAGTAGGTCT	SNP Confirmation
3′ BT0747 SNP Diag	GGAACACCTCACAATTCCCCT	SNP Confirmation

a Relevant restriction sites are indicated by bold lettering.

b YakYel, 5′ Yakima yellow dye; ZEN, ZEN internal quencher; IABkFQ, Iowa Black FQ 3′ quencher.

Allelic exchange mutagenesis was used for generation of the BtΔ*cdaA* mutant and subsequent complementation. To make the Δ*cdaA* mutational construct, 5′ (primers 5′ F1 *bt0008* KO and 3′ F1 *bt0008* KO_AscI) and 3′ (primers 5′ F2 *bt0008* KO_AscI and 3′ F2 *bt0008* KO_BssHII) flanking regions were amplified from BtWT gDNA. These flanking regions were then ligated together with the B. turicatae-adapted gentamicin resistance cassette from pUAMS4 between them, generating the Δ*cdaA* construct pUAMS248B (89). In the final mutational construct, the *aacC1* marker replaces a 419-bp internal region between nucleotides 86 and 504 of the *bt0008* ORF. For complementation, a B. turicatae-adapted kanamycin resistance cassette was made by amplifying the putative promoter region for the *flaB* gene from BtWT gDNA (primers 5′ BtFlaB-*aphI*-BamHI and 3′ Kan ORF int -NdeI v.2) and the Tn*903*-derived aminoglycoside phosphotransferase ORF (*aphI*) from pBSV2 (primers 5′ Kan ORF int -NdeI v.2 and 3′ BtFlaB-*aphI*-BamHI) (153). These amplicons were then fused together by overlap extension PCR, and the resulting product was ligated into pGEM-T Easy, generating the plasmid pUAMS309. To create the Δ*cdaA^C-cis^* complementation construct, a segment encoding the 5′ flanking region and disrupted *cdaA* ORF (primers 5′ F1 *bt0008* KO and 5′ Bt*flgB*-BamHI) was amplified from pUAMS248B, and a segment encoding the intact *cdaA* ORF with its adjacent upstream region, as well as a downstream segment to facilitate recombination (primers 5′ F2 *bt0008* Comp-BamHI and 3′ F2 *bt0008* Comp-BglII), was amplified from BtWT gDNA. These segments were then ligated together with the P*flaB*-*aphI* resistance cassette from pUAMS309 between them, yielding the final Δ*cdaA^C-cis^* construct, pUAMS313B.

To complement the BtΔ*cdaA* mutant with a mutated copy of *cdaA* containing a point mutation converting a glycine at position 175 to an alanine, an approach similar to the BtΔ*cdaA^C-cis^* complementation strategy was used. To introduce the G175A point mutation into *cdaA*, overlap extension PCR was performed with pUAMS313B serving as the template (primer pairs 5′ DAC/*cis bt0008* and 3′ *bt0008* G175A; 5′ *bt0008* G175A and 3′ DAC/*cis bt0008*). The full-length product was then ligated into pUAMS313B, replacing the analogous region and yielding the Δ*cdaA^C-cis^*^(G175A)^ construct, pUAMS351B.

For complementation of BtΔ*cdaA^C-cis^*^(G175A)^, a B. turicatae-adapted *aadA* streptomycin resistance cassette was first made by amplifying the putative promoter region for the *flaB* gene from BtWT gDNA (primers 5′ BtP*flaB*_BamHI and 3′ P*flaB* NdeI) and the aminoglycoside adenylyltransferase ORF (*aadA*) from pJD7 (primers 5′ Bt*flaB*-*aadA* junc_NdeI and 3′ *aadA* ORF_BamHI) (154). The promoter and *aadA* ORF were fused by ligating the two amplicons together using the common NdeI restriction site. The final ligated P*flaB*-*aadA* cassette in pGEM-T Easy was designated pUAMS402. The P*flaB*-*aadA* cassette was subsequently ligated into pUAMS313B in place of the P*flaB*-*aphI* cassette using the BamHI sites, generating the final Δ*cdaA^C-cis^*^(G175A)^::*cdaA^C-cis^*^(WT)^ construct pUAMS406B.

To generate the B. turicatae shuttle vector, pBtSV-JB, a region capable of autonomous replication in B. turicatae (BtOri), based on homology to B. burgdorferi and B. hermsii shuttle vectors, was assembled with an E. coli pUC/pMB1 *ori* and the *aphI* gene expressed from the B. turicatae *flaB* promoter (152, 153, 155). The pUC/pMB1 *ori* was amplified from the B. burgdorferi shuttle vector pJD44 (primers 5′ pJD44 ColE1 *ori* and 3′ pJD44 ColE1 *ori*). The BtOri region was then amplified from BtWT gDNA (primers 5′ UAMS BtOri BamHI and 3′ UAMS BtOri AscI). Next, the P*flaB*-*aphI* kanamycin resistance marker was amplified from pUAMS309 (primers 5′ UAMS-88 Kan BglII and 3′ UAMS-88 Kan AscI) and ligated into pJD44 to fuse the pJD44 multiple cloning site (MCS) to P*flaB*-*aphI* (pJD44::P*flaB*-*aphI*). pBtSV-JB was assembled by excising the MCS-P*flaB*-*aphI* fusion from the pJD44::P*flaB*-*aphI* intermediate with AscI and ligating with the BglII/AscI-digested ColE1 fragment and BamHI/AscI-digested BtOri region.

The *lac*-inducible expression constructs were generated in the backbone of pBtSV-JB (above). The region containing the MCS and *lac*-inducible promoter Bb*luc+* of pJSB104 was moved into pBtSV-JB using BamHI and HindIII (154). For overproduction of LacI, the *Borrelia* codon-adapted BbLacI was expressed from the B. turicatae flagellar basal body rod protein (*flgB*) promoter. The *flgB* promoter was amplified from BtWT gDNA (primers 5′ Bt-P*flgB*/BamHI-HindIII and 3′ Bt-P*flgB*/BbLacI junc), and the BbLacI ORF was amplified from pJSB104 (primers 5′ Bt-P*flgB*/BbLacI junc and 3′ Bt-BbLacI/BglII). These amplicons were then fused together by overlap extension PCR, and the resulting product was ligated into pGEM-T Easy. For the construct used to overexpress *rpoS* and *cdaA*, the P*flgB*-Bb*lacI* fusion was excised with BamHI and BglII and then ligated into BglII-digested pBtSV-JB. For the construct used to overexpress *bosR*, the P*flgB*-Bb*lacI* fusion was excised with BamHI and BglII and then ligated into BamHI-digested pBtSV-JB. Next, the *rpoS*, *cdaA*, and *bosR* ORFs were amplified from BtWT gDNA with the following primer pairs: 5′ BtRpoS ORF-NdeI and 3′ BtRpoS ORF-HindIII, 5′ *bt0008* ORF NdeI and 3′ *bt0008* ORF HindIII, and 5′ BtBosR ORF NdeI and 3′ BtBosR ORF HindIII, respectively. These ORF regions were digested with NdeI and HindIII and ligated into the inducible expression constructs digested with the same enzymes to generate pUAMS267A (piRpoS), pUAMS346 (piCdaA), and pUAMS446B (piBosR), respectively.

### Transformation of B. turicatae, clonal isolation, and genotypic confirmation.

Electroporation of B. turicatae was performed as previously described (156). Briefly, the mutagenesis construct, complementation construct, or shuttle vector of interest was electroporated into the appropriate strain. Following a 24-h recovery period in mBSK medium without selection, transformants were selected for antibiotic treatment. Clones were then isolated by serial dilution plating. All confirmatory PCRs herein were separated by electrophoresis in a 0.8% agarose gel and visualized by ethidium bromide staining. GeneRuler DNA Ladder mix (Thermo Fisher Scientific, Waltham, MA) served as the molecular weight standard. To confirm shuttle vector-transformed clones, plasmids were recovered by transformation into E. coli, analyzed by restriction digest, and Sanger sequenced (data not shown).

To confirm BtΔ*cdaA* mutant and BtΔ*cdaA^C-cis^* clones, PCRs were performed to amplify a region within the replaced segment of *cdaA* (primers 5′ *bt0008* int diag and 3′ *bt0008* int diag), an internal segment of *aacC1* (primers 5′ *aacC1* diag and 3′ *aacC1* diag), an internal region of *aphI* (primers 5′ *aphI* diag and 3′ *aphI* diag), and an internal segment of *flaB* as an amplification control (primers 5′ BtFlaB and 3′ BtFlaB). To confirm complementation of the BtΔ*cdaA* strain with the mutated *cdaA* ORF [BtΔ*cdaA^C-cis^*^(G175A)^] and subsequent complementation with the wild-type copy of *cdaA* [BtΔ*cdaA^C-cis^*^(G175A)^::*cdaA^C-cis^*^(WT)^], PCRs were conducted to amplify an internal segment of *cdaA* (see above), an internal segment of *aphI* (see above), an internal segment of *aadA* (primers 5′ *aadA* diag and 3′ *aadA* diag), and an internal segment of *flaB* (see above). A region of *cdaA* containing the point mutation was additionally amplified (primers 5′ *bt0008* int diag and 3′ *bt0008* ext diag), gel purified, and Sanger sequenced to confirm the G175A mutation.

The *cdaA* conditional mutant (BtiCdaA-Δ*cdaA*) was generated similar to previously described methods in B. burgdorferi (99, 100). BtWT was first transformed with pUAMS346 to generate BtiCdaA. A confirmed BtiCdaA clone was then transformed with the Δ*cdaA* mutational construct pUAMS248B, and bacteria were recovered after electroporation in the presence of 1 mM IPTG to maintain expression of *cdaA* in spirochetes that acquired the mutation. Following the 24-h recovery period, bacteria were passed into media containing kanamycin, gentamicin, and 1 mM IPTG to select for bacteria with the Δ*cdaA* mutation. BtiCdaA-Δ*cdaA* clones were then isolated using serial dilution plating (156). The conditional mutant was confirmed to harbor pUAMS346 by plasmid recovery in E. coli, and the mutation was confirmed by differential PCR using primers flanking the mutation (primers 5′ *bt0008* ext diag and 3′ *bt0008* ext diag), as well as PCRs for internal segments of the *aacC1* marker (see above) and *flaB* gene (see above).

### Expression and purification of recombinant protein.

Expression from pProEX-HTb generates recombinant protein with an N-terminal His_6_ tag and tobacco etch virus (TEV) cleavage site. pUAMS338 was transformed into C41(DE3) E. coli, and expression of recombinant CdaA lacking the transmembrane domains was achieved by induction with 1 mM IPTG for 3 h at 37°C. For expression of recombinant truncated RpoS, pUAMS233 was also transformed into C41(DE3) E. coli, and expression was induced with 1 mM IPTG for 3 h at 37°C. For expression of recombinant BosR, pUAMS159 was cotransformed into C41(DE3) E. coli with pRARE (Novagen, Madison, WI), a plasmid isolated from RosettaBlue(DE3) cells that encodes tRNAs to express genes with rare E. coli codons (157). Expression of recombinant BosR was then achieved by induction with 1 mM IPTG for 3 h at 37°C. Recombinant CdaA, RpoS, and BosR were purified using HisPur nickel-nitrilotriacetic acid (Ni-NTA) resin (Thermo Fisher Scientific) under nonnative conditions. Briefly, cells were lysed using BugBuster protein extraction reagent (MilliporeSigma, Burlington, MA) in conjunction with Lysonase (MilliporeSigma) per the manufacturer’s instructions. Following cell lysis, soluble and insoluble fractions were separated by centrifugation at 24,000 × *g* for 15 min. Inclusion bodies were then solubilized in resin wash buffer (20 mM Tris, 20 mM NaCl, 5% glycerol, pH 7.5) supplemented with 0.3% N-lauryl-sarcosine. Ni-NTA resin was washed two times in this same buffer prior to combining with the solubilized inclusion body. Binding was then performed using an end-over-end rotator for >30 min. Following binding, the resin was washed in 10 bed volumes of buffer A (20 mM Tris, 20 mM NaCl, 20 mM imidazole, and 5% glycerol, pH 7.5) supplemented with 0.3% N-lauryl-sarcosine, followed by a wash in 10 resin bed volumes of buffer B (20 mM Tris, 1 M NaCl, and 5% glycerol, pH 7.5) supplemented with 0.3% N-lauryl-sarcosine. A final wash was then performed in 10 resin bed volumes of buffer A with 0.3% N-lauryl-sarcosine. Finally, the protein was eluted from the resin using buffer C (20 mM Tris, 200 mM NaCl, 250 mM imidazole, and 5% glycerol, pH 7.5) with 0.3% N-lauryl-sarcosine. Following purification, concentrations of purified recombinant proteins were calculated using the DC protein assay kit (Bio-Rad Laboratories, Hercules, CA).

### Generation of CdaA-, RpoS-, and BosR-specific antiserum.

Rat immunizations were performed in accordance with the recommendations of the Public Health Science (PHS) Policy on Humane Care and Use of Laboratory Animals, the Guide for the Care and Use of Laboratory Animals, and the Animal Welfare Act, and the utilized protocol was approved by the University of Arkansas for Medical Sciences (UAMS) Institutional Animal Care and Use Committee (IACUC). Generation of CdaA-, RpoS-, and BosR-specific rat antiserum was performed as previously described (158). Briefly, 25 μg of recombinant protein in 200 μl of phosphate-buffered saline (PBS) was emulsified with 200 μl of complete Freund’s adjuvant (Sigma-Aldrich, St. Louis, MO) and intraperitoneally injected into 3- to 4-week-old female Sprague-Dawley rats (Envigo, Indianapolis, IN, and Charles River Laboratories, Wilmington, MA). Rats were subsequently boosted twice at 4-week intervals by intraperitoneal injection of 25 μg of recombinant protein in 200 μl of PBS emulsified with 200 μl of incomplete Freund’s adjuvant (Sigma-Aldrich). Two weeks following the final boost, rats were euthanized, and serum was collected.

### SDS-PAGE and immunoblotting.

Immunoblot analyses were performed as previously described (159). Briefly, whole-cell lysates were prepared from B. turicatae cultures grown to late exponential phase. A volume of lysate equivalent to 2 × 10^7^ spirochetes was then separated by SDS-PAGE and transferred to a nitrocellulose membrane. Membranes were subsequently probed with rat antiserum recognizing CdaA, BosR, or RpoS (see above). Horseradish peroxidase (HRP)-conjugated goat anti-rat IgG (Jackson ImmunoResearch Laboratories, West Grove, PA) was used as a secondary antibody. As a positive control, membranes were additionally probed for FlaB using chicken anti-B. burgdorferi FlaB IgY as the primary antibody and HRP-conjugated donkey anti-chicken IgY (Jackson ImmunoResearch Laboratories) as a secondary antibody (159). To assess seroconversion of mice from murine infection experiments (see below), BtWT lysates were probed with serum from infected or naive mice, and HRP-conjugated goat anti-mouse IgG (Jackson ImmunoResearch Laboratories) served as the secondary antibody (data not shown). For all blots, colorimetric detection was achieved using 4-chloro-1-naphthol as a substrate, and Precision Plus Protein all blue prestained protein standard (Bio-Rad Laboratories) served as the molecular weight standard.

### Murine infection experiments.

Murine infection experiments were performed in accordance with the recommendations of the PHS Policy on Humane Care and Use of Laboratory Animals, the Guide for the Care and Use of Laboratory Animals, and the Animal Welfare Act, and the utilized protocol was approved by the UAMS IACUC. Infections were performed as previously described (89). Briefly, 4- to 6-week-old female Swiss Webster mice (Charles River Laboratories) were intradermally/subcutaneously injected with 10^2^ bacteria in the thoracic region. On days 3 to 14 postinfection, 2.5 μl of blood was taken by tail vein venipuncture, combined with 47.5 μl of SideStep lysis and stabilization buffer (Agilent Technologies, Santa Clara, CA), and stored at −80°C until qPCR analyses were performed to quantify bacteremia (see below). An additional 2.5 μl of blood was then collected and added to mBSK medium supplemented with *Borrelia* antibiotic mixture (BAM) (Monserate, San Diego, CA) to assess presence of live bacteria in the bloodstream. On day 14, mice were euthanized, blood was collected by brachial artery bleed, and serum was isolated for immunoblot analyses for seroconversion. Two weeks following collection of daily blood samples, cultures were assessed for presence of spirochetes; 10 fields of view were scanned by dark-field microscopy, and presence of one or more spirochetes was considered culture positive. Given the volume of blood taken during the murine infection experiments, the LOD for this analysis is equivalent to one spirochete/2.5 μl of blood (4 × 10^2^ bacteria/ml). For use in qPCR standards, naive mice were euthanized, and blood was collected by brachial artery bleed and added to lysis and stabilization buffer at a blood-to-buffer ratio of 1:18. Blood/buffer mixtures from naive mice were stored at −80°C until standard preparation (see below).

### qPCR for bacterial burdens.

qPCR analyses to detect spirochetemia in mice were performed as previously described (89). Briefly, in a 96-well real-time PCR plate, 3 μl of blood in lysis and stabilization buffer (see above) was added in a 20-μl reaction mixture containing 10 μl of SsoAdvanced universal probes supermix (Bio-Rad Laboratories) and final concentrations of 400 nM and 300 nM for primers (Bt*flaB* F and Bt*flaB* R) and probe (Bt*flaB*-Probe), respectively. For qPCR standards, late-exponential BtWT cultures were pelleted, washed twice in PBS supplemented with 5 mM MgCl_2_ (PBS-MgCl_2_), and then resuspended in PBS-MgCl_2_ and quantified by dark-field microscopy. This suspension was then used to make serial dilutions in PBS-MgCl_2_ from 10^4^ to 10^8^ bacteria/ml. For use in the no-template control (NTC), nuclease-free water was diluted 10-fold in PBS-MgCl_2_. These dilutions were then added to naive blood in lysis and stabilization buffer (see above) at a 1:19 ratio, and 3 μl of these preparations were added in a 20-μl reaction mixture, as described for murine blood samples above, to generate a standard curve. qPCRs for all samples and standards were performed in triplicate. The QuantStudio 6 Flex real-time PCR system (Thermo Fisher Scientific) was used for real-time PCR, and the reaction conditions consisted of an initial 2 min, 50°C hold followed by a 10 min, 95°C polymerase activation step. Forty cycles of DNA denaturation at 95°C for 15 s and primer annealing/DNA extension at 60°C for 60 s were then performed for DNA amplification. Data were then imported into GraphPad Prism version 8 (GraphPad Software, San Diego, CA) for analysis.

### qRT-PCR analyses.

RNA extraction, cDNA synthesis, and qRT-PCR were performed as previously described (89, 154, 158). Briefly, B. turicatae cultures were grown to late exponential growth phase followed by addition of 10% RNA stop solution (154, 160). Bacteria were then collected by centrifugation and stored at −80°C until RNA isolation was performed. RNA was isolated by TRIzol extraction (Thermo Fisher Scientific), followed by purification with the RNeasy minikit (Qiagen, Valencia, CA) according to the manufacturer's instructions. Treatment with RNase-free DNase I (Qiagen) was then performed to degrade possible contaminating DNA, and absence of B. turicatae gDNA was confirmed using PCR to amplify an internal segment of the *flaB* gene (see above). The iScript cDNA synthesis kit (Bio-Rad Laboratories) was then used to reverse transcribe purified RNA into cDNA via the manufacturer’s protocol. As a negative control, mock reactions were performed in the absence of reverse transcriptase. Successful cDNA synthesis was then confirmed by PCR amplification of an internal segment of the *flaB* gene (see above).

qRT-PCR was used to measure expression of *bt0007* (primers S-*bt0007*_IDT-SYBR and AS-*bt0007*_IDT-SYBR), *cdaA* (primers S-*bt0008*_IDT-SYBR_V2 and AS-*bt0008*_IDT-SYBR_V2), *bt0009* (primers S-*bt0009*_IDT-SYBR and AS-*bt0009*_IDT-SYBR), *bt0010* (primers 2_*bt0010*_IDT-SYBR FWD and 2_*bt0010*_IDT-SYBR REV), *bosR* (primers *bt0647* SYBR/IDT FWD Set 1 and *bt0647* SYBR/IDT REV Set 1), *rpoS* (primers Bt*rpoS* SYBR FWD and Bt*rpoS* SYBR REV), and *flaB* (primers 5′ Bt*flaB* SYBR/ABI and 3′ Bt*flaB* SYBR/ABI). SYBR reactions were performed with SsoAdvanced universal SYBR green supermix (Bio-Rad Laboratories) per the manufacturer's instructions with 100 ng of cDNA serving as the template, and all reactions were performed in triplicate. The QuantStudio 6 Flex real-time PCR system was used for real-time PCR with reaction conditions consisting of an initial polymerase activation step at 95°C for 30 s and 40 cycles of DNA denaturation at 95°C for 10 s and primer annealing/DNA extension at 60°C for 30 s. Results were imported into GraphPad Prism version 8 for analysis.

### Whole-genome sequencing and SNP analysis.

For WGS analyses, B. turicatae strains of interest were grown to late exponential growth phase, and DNA was purified using the DNeasy blood and tissue kit (Qiagen) via the manufacturer’s instructions. gDNA quality was then analyzed using the TapeStation 2200 system (Agilent Technologies), and only samples with DNA integrity numbers (DINs) above 8 were utilized. The Nextera XT DNA library preparation kit (Illumina Inc., San Diego, CA) was used for library preparation, and the MiSeq system (Illumina Inc.) was used for next-generation sequencing using 500-cycle, 2 × 250-bp chemistry. The paired-end FASTQ files generated were processed using fastp (v0.20.0) for quality filtering (Phred score ≥20), adapter trimming, and read correction (161). All sequencing data sets had a coverage of approximately ×400 to ×500 postfiltering and correction. Variant analysis was performed using an “all-in-one” variant calling pipeline, Snippy (v4.6.0), using default parameters (–mapqual 60, –basequal 13, –mincov 10, –minqual 100) with –cpus 30 (162). The updated, complete B. turicatae 91E135 genome generated by the Job Lopez laboratory at Baylor College of Medicine was used as the reference (J. E. Lopez and A. R. Kneubehl, unpublished data). BtWT was used to assess any differences between the B. turicatae 91E135 from our lab and what was sequenced by the Lopez laboratory. This analysis yielded only eight SNPs/variants between BtWT and the Lopez B. turicatae 91E135 genome. Variants were then assessed in BtΔ*cdaA* clones 1 and 2 using the same reference genome. The variants reported herein were those found to be different from the BtWT variants, indicating differences between the mutant clones and their parental cell line compared to the reference genome. The variant analyses were performed on a System76 Thelio Massive Linux machine with an Intel Xeon Gold 6230 processor and 126 Gb ECC DDR4 2933 MHz RAM (System76, Denver, CO). All SNPs were confirmed by amplifying a segment containing the SNP, gel purifying the product, and Sanger sequencing. The following primer pairs were used for amplification of the regions containing the SNPs in *bt0380*, *bt0531*, *bt0127*, *bt0219*, *bt0241B*, and *bt0747*: 5′ BT0380 SNP Diag and 3′ BT0380 SNP Diag, 5′ BT0531 SNP Diag and 3′ BT0531 SNP Diag, 5′ BT0127 SNP Diag and 3′ BT0127 SNP Diag, 5′ BT0219 SNP Diag and 3′ BT0219 SNP Diag, 5′ BT0241B SNP Diag and 3′ BT0241B SNP Diag, and 5′ BT0747 SNP Diag and 3′ BT0747 SNP Diag, respectively.

### Quantification of c-di-AMP.

For quantification of c-di-AMP, the B. turicatae strains of interest were grown to late exponential growth phase, and 10^10^ cells were pelleted and washed two times in filtered saline. Following the last wash, cells were resuspended in 50 μl of Milli-Q H_2_O, heat inactivated for 10 min at 99°C, and stored at −80°C until c-di-AMP quantification was performed. Cell resuspensions were mixed with 25 μM heavy (C^13^N^15^) c-di-AMP, followed by 500 μl of methanol, and sonicated (20 s, 80% amplitude). Methanol was collected following centrifugation of cell lysates. The remaining cell lysates were then resuspended in 50 μl Milli-Q H_2_O, mixed with 500 μl methanol, and sonicated (10 s, 80% amplitude) again. The second methanol fraction was pooled with the first following centrifugation. Fractions were dried in a speed vacuum concentrator, and resultant pellets were resuspended in 30 μl Milli-Q H_2_O for mass spectrometry analysis. Quantification of c-di-AMP was based on the 659/136 mass transition (689/146 for the internal standard) as previously described (68).

### Growth curve analyses.

For *in vitro* growth curve analyses of BtWT, BtΔ*cdaA^C-cis^*^(G175A)^, and BtΔ*cdaA^C-cis^*^(G175A)^::*cdaA^C-cis^*^(WT)^, cultures were inoculated at a density of 10^4^ spirochetes/ml in normal mBSK medium or in the following conditions: (i) mBSK medium supplemented with 50 mM NaCl or 50 mM KCl; (ii) mBSK medium diluted 1:10 in 1× PBS, 1.25× PBS, or 1.50× PBS; or (iii) mBSK medium prepared without pyruvate. For *in vitro* growth curve analysis of BtiCdaA-Δ*cdaA*, an initial 1-ml starter culture was grown to late exponential growth phase in mBSK medium supplemented with 1 mM IPTG, followed by centrifugation at 5,283 × *g* for 5 min and resuspension in 1 ml mBSK without IPTG supplementation. This wash step was repeated twice to remove any remaining IPTG from the culture. Following the washes, BtiCdaA-Δ*cdaA* was inoculated into media with or without 1 mM IPTG at an initial density of 10^3^ bacteria/ml. On the indicated days postinoculation, bacteria were quantified by dark-field microscopy. Ten microscopic fields were counted for each biological replicate, and two biological replicates for each condition were analyzed.

### Imaging of *Borrelia* spirochetes and quantification of membrane blebbing.

BtiCdaA-Δ*cdaA* was grown in the presence or absence of 1 mM IPTG to late exponential growth phase, centrifuged at 9,391 × *g* for 5 min, and resuspended in PBS-MgCl_2_. This step was repeated one time, and the washed spirochetes in PBS-MgCl_2_ were used for microscopy. To immobilize the spirochetes for visualization and imaging, bacterial resuspensions were spotted onto a 1% agarose pad on a microscope slide and covered with a coverslip (163). The Keyence BZ-X800 microscope (Keyence Corp., Itasca, IL) was used to visualize bacteria and capture bright-field images at ×60 magnification with oil immersion. Two biological replicates were analyzed, and 100 spirochetes from each replicate were assessed for membrane blebbing.

### Statistical methods.

All statistical analyses were performed using GraphPad Prism version 8. Two-way analysis of variance (ANOVA) models with Dunnett’s test for multiple comparisons were used to compare growth of mutant and complemented strains to the BtWT parent. To compare growth of BtiCdaA-Δ*cdaA* grown with and without IPTG, a two-way ANOVA model with Bonferroni’s test for multiple comparisons was used. For comparison of numbers of bacteria exhibiting membrane blebbing in BtiCdaA-Δ*cdaA* grown with and without IPTG, an unpaired Student's *t* test was used. *P* values of 0<0.05 were considered statistically significant. For qRT-PCR analyses, the threshold cycle (2^−ΔΔ^*^CT^*) method was used to calculate fold change in gene expression relative to BtWT as previously described (89, 164).

## References

[1] Ross PH, Milne AD. 1904. Tick fever. Br Med J 2:1453–1454. 10.1136/bmj.2.2291.1453. 20761784 PMC2355890

[2] Dutton JE, Todd JL, Newstead R. 1905. The nature of human tick-fever in the eastern part of the Congo free-state, p 4–26. Pub. for the University Press of Liverpool by Williams & Norgate, London, UK.

[3] Cutler SJ, Abdissa A, Trape JF. 2009. New concepts for the old challenge of African relapsing fever borreliosis. Clin Microbiol Infect 15:400–406. 10.1111/j.1469-0691.2009.02819.x. 19489922

[4] Cutler SJ. 2010. Relapsing fever–a forgotten disease revealed. J Appl Microbiol 108:1115–1122. 10.1111/j.1365-2672.2009.04598.x. 19886891

[5] Dworkin MS, Anderson DE, Jr, Schwan TG, Shoemaker PC, Banerjee SN, Kassen BO, Burgdorfer W. 1998. Tick-borne relapsing fever in the northwestern United States and southwestern Canada. Clin Infect Dis 26:122–131. 10.1086/516273. 9455520

[6] Dworkin MS, Schwan TG, Anderson DE, Jr, Borchardt SM. 2008. Tick-borne relapsing fever. Infect Dis Clin North Am 22:449–468. 10.1016/j.idc.2008.03.006. 18755384 PMC3725823

[7] Stoenner HG, Dodd T, Larsen C. 1982. Antigenic variation of *Borrelia hermsii*. J Exp Med 156:1297–1311. 10.1084/jem.156.5.1297. 7130900 PMC2186838

[8] Cadavid D, Thomas DD, Crawley R, Barbour AG. 1994. Variability of a bacterial surface protein and disease expression in a possible mouse model of systemic Lyme borreliosis. J Exp Med 179:631–642. 10.1084/jem.179.2.631. 8294872 PMC2191368

[9] Pennington PM, Allred CD, West CS, Alvarez R, Barbour AG. 1997. Arthritis severity and spirochete burden are determined by serotype in the *Borrelia turicatae*-mouse model of Lyme disease. Infect Immun 65:285–292. 10.1128/IAI.65.1.285-292.1997. 8975925 PMC174589

[10] Jongen VH, van Roosmalen J, Tiems J, Van Holten J, Wetsteyn JC. 1997. Tick-borne relapsing fever and pregnancy outcome in rural Tanzania. Acta Obstet Gynecol Scand 76:834–838. 10.3109/00016349709024361. 9351408

[11] Centers for Disease Control and Prevention. 2007. Acute respiratory distress syndrome in persons with tickborne relapsing fever–three states, 2004-2005. MMWR Morb Mortal Wkly Rep 56:1073–1076. 17947965

[12] Parry EH, Warrell DA, Perine PL, Vukotich D, Bryceson AD. 1970. Some effects of louse-borne relapsing fever on the function of the heart. Am J Med 49:472–479. 10.1016/S0002-9343(70)80041-9. 4249048

[13] Vial L, Diatta G, Tall A, Ba el H, Bouganali H, Durand P, Sokhna C, Rogier C, Renaud F, Trape JF. 2006. Incidence of tick-borne relapsing fever in west Africa: longitudinal study. Lancet 368:37–43. 10.1016/S0140-6736(06)68968-X. 16815378

[14] Dupont HT, La Scola B, Williams R, Raoult D. 1997. A focus of tick-borne relapsing fever in southern Zaire. Clin Infect Dis 25:139–144. 10.1086/514496. 9243047

[15] McConnell J. 2003. Tick-borne relapsing fever under-reported. Lancet Infect Dis 3:604. 10.1016/S1473-3099(03)00787-4. 14558501

[16] Davis GE. 1936. *Ornithodoros turicata*: the possible vector of relapsing fever in southwestern Kansas: preliminary report. Pub Health Rep 51:1719. 10.2307/4582025.

[17] Davis GE. 1941. *Ornithodoros hermsi* and relapsing fever in Oregon. Pub Health Rep 56:2010–2012. 10.2307/4583889.

[18] Davis GE, Mazzotti L. 1953. The non-transmission of the relapsing fever spirochete, *Borrelia dugesii* (Mazzotti, 1949) by the argasid tick *Ornithodoros turicata* (Dugès, 1876). J Parasitol 39:663–666. 10.2307/3274086. 13118437

[19] Kemp HA, Moursund WH, Wright HE. 1934. Relapsing fever in Texas II. The specificity of the vector, *Ornithodorus turicata*, for the spirochete. Am J Trop Med Hyg s1-14:159–162. 10.4269/ajtmh.1934.s1-14.159.

[20] Warrell DA. 2019. Louse-borne relapsing fever (*Borrelia recurrentis* infection). Epidemiol Infect 147:e106. 10.1017/S0950268819000116. 30869050 PMC6518520

[21] Boutellis A, Abi-Rached L, Raoult D. 2014. The origin and distribution of human lice in the world. Infect Genet Evol 23:209–217. 10.1016/j.meegid.2014.01.017. 24524985

[22] Raoult D, Roux V. 1999. The body louse as a vector of reemerging human diseases. Clin Infect Dis 29:888–911. 10.1086/520454. 10589908

[23] Ciervo A, Mancini F, di Bernardo F, Giammanco A, Vitale G, Dones P, Fasciana T, Quartaro P, Mazzola G, Rezza G. 2016. Louseborne relapsing fever in young migrants, Sicily, Italy, July-September 2015. Emerg Infect Dis 22:152–153. 10.3201/eid2201.151580. 26690334 PMC4696720

[24] Hoch M, Wieser A, Löscher T, Margos G, Pürner F, Zühl J, Seilmaier M, Balzer L, Guggemos W, Rack-Hoch A, von Both U, Hauptvogel K, Schönberger K, Hautmann W, Sing A, Fingerle V. 2015. Louse-borne relapsing fever (*Borrelia recurrentis*) diagnosed in 15 refugees from northeast Africa: epidemiology and preventive control measures, Bavaria, Germany, July to October 2015. Euro Surveill 20. 10.2807/1560-7917.es.2015.20.42.30046. 26538532

[25] European Centre for Disease Prevention and Control. 2015. Rapid risk assessment: louse-borne relapsing fever in the EU. European Centre for Disease Prevention and Control, Stockholm, Sweden.

[26] Antinori S, Mediannikov O, Corbellino M, Raoult D. 2016. Louse-borne relapsing fever among East African refugees in Europe. Travel Med Infect Dis 14:110–114. 10.1016/j.tmaid.2016.01.004. 26872415

[27] Hytönen J, Khawaja T, Grönroos JO, Jalava A, Meri S, Oksi J. 2017. Louse-borne relapsing fever in Finland in two asylum seekers from Somalia. APMIS 125:59–62. 10.1111/apm.12635. 27859692

[28] Ackermann N, Marosevic D, Hörmansdorfer S, Eberle U, Rieder G, Treis B, Berger A, Bischoff H, Bengs K, Konrad R, Hautmann W, Schönberger K, Belting A, Schlenk G, Margos G, Hoch M, Pürner F, Fingerle V, Liebl B, Sing A. 2018. Screening for infectious diseases among newly arrived asylum seekers, Bavaria, Germany, 2015. Euro Surveill 23:17-00176. 10.2807/1560-7917.es.2018.23.10.17-00176. PMC5850590 29536830

[29] Isenring E, Fehr J, Gültekin N, Schlagenhauf P. 2018. Infectious disease profiles of Syrian and Eritrean migrants presenting in Europe: a systematic review. Travel Med Infect Dis 25:65–76. 10.1016/j.tmaid.2018.04.014. 29702253

[30] Gomelsky M. 2011. cAMP, c-di-GMP, c-di-AMP and now cGMP: bacteria use them all! Mol Microbiol 79:562–565. 10.1111/j.1365-2958.2010.07514.x. 21255104 PMC3079424

[31] Kalia D, Merey G, Nakayama S, Zheng Y, Zhou J, Luo Y, Guo M, Roembke BT, Sintim HO. 2013. Nucleotide, c-di-GMP, c-di-AMP, cGMP, cAMP, (p)ppGpp signaling in bacteria and implications in pathogenesis. Chem Soc Rev 42:305–341. 10.1039/c2cs35206k. 23023210

[32] Corrigan RM, Gründling A. 2013. Cyclic di-AMP: another second messenger enters the fray. Nat Rev Microbiol 11:513–524. 10.1038/nrmicro3069. 23812326

[33] Fahmi T, Port GC, Cho KH. 2017. c-di-AMP: an essential molecule in the signaling pathways that regulate the viability and virulence of Gram-positive bacteria. Genes (Basel) 8:197. 10.3390/genes8080197. PMC5575661 28783096

[34] Commichau FM, Heidemann JL, Ficner R, Stulke J. 2018. Making and breaking of an essential poison: the cyclases and phosphodiesterases that produce and degrade the essential second messenger cyclic di-AMP in bacteria. J Bacteriol 201:e00462-18. 10.1128/JB.00462-18. 30224435 PMC6287462

[35] Huynh TN, Woodward JJ. 2016. Too much of a good thing: regulated depletion of c-di-AMP in the bacterial cytoplasm. Curr Opin Microbiol 30:22–29. 10.1016/j.mib.2015.12.007. 26773214 PMC4821758

[36] Zarrella TM, Bai G. 2020. The many roles of the bacterial second messenger cyclic di-AMP in adapting to stress cues. J Bacteriol 203:e00348-20. 10.1128/JB.00348-20. 32839175 PMC7723955

[37] Bowman L, Zeden MS, Schuster CF, Kaever V, Grundling A. 2016. New insights into the cyclic di-adenosine monophosphate (c-di-AMP) degradation pathway and the requirement of the cyclic dinucleotide for acid stress resistance in *Staphylococcus aureus*. J Biol Chem 291:26970–26986. 10.1074/jbc.M116.747709. 27834680 PMC5207132

[38] Witte CE, Whiteley AT, Burke TP, Sauer JD, Portnoy DA, Woodward JJ. 2013. Cyclic di-AMP is critical for *Listeria monocytogenes* growth, cell wall homeostasis, and establishment of infection. mBio 4:e00282-13. 10.1128/mBio.00282-13. 23716572 PMC3663569

[39] Zarrella TM, Metzger DW, Bai G. 2018. Stress suppressor screening leads to detection of regulation of cyclic di-AMP homeostasis by a Trk family effector protein in *Streptococcus pneumoniae*. J Bacteriol 200:e00045-18. 10.1128/JB.00045-18. 29483167 PMC5971481

[40] Luo Y, Helmann JD. 2012. Analysis of the role of *Bacillus subtilis* σ(M) in β-lactam resistance reveals an essential role for c-di-AMP in peptidoglycan homeostasis. Mol Microbiol 83:623–639. 10.1111/j.1365-2958.2011.07953.x. 22211522 PMC3306796

[41] Cheng X, Zheng X, Zhou X, Zeng J, Ren Z, Xu X, Cheng L, Li M, Li J, Li Y. 2016. Regulation of oxidative response and extracellular polysaccharide synthesis by a diadenylate cyclase in *Streptococcus mutans*. Environ Microbiol 18:904–922. 10.1111/1462-2920.13123. 26548332

[42] Dengler V, McCallum N, Kiefer P, Christen P, Patrignani A, Vorholt JA, Berger-Bächi B, Senn MM. 2013. Mutation in the c-di-AMP cyclase *dacA* affects fitness and resistance of methicillin resistant *Staphylococcus aureus*. PLoS One 8:e73512. 10.1371/journal.pone.0073512. 24013956 PMC3754961

[43] Smith WM, Pham TH, Lei L, Dou J, Soomro AH, Beatson SA, Dykes GA, Turner MS. 2012. Heat resistance and salt hypersensitivity in *Lactococcus lactis* due to spontaneous mutation of llmg_1816 (*gdpP*) induced by high-temperature growth. Appl Environ Microbiol 78:7753–7759. 10.1128/AEM.02316-12. 22923415 PMC3485701

[44] Fahmi T, Faozia S, Port GC, Cho KH. 2019. The second messenger c-di-AMP regulates diverse cellular pathways involved in stress response, biofilm formation, cell wall homeostasis, SpeB expression, and virulence in *Streptococcus pyogenes*. Infect Immun 87:e00147-19. 10.1128/IAI.00147-19. 30936159 PMC6529668

[45] Cho KH, Kang SO. 2013. Streptococcus pyogenes c-di-AMP phosphodiesterase, GdpP, influences SpeB processing and virulence. PLoS One 8:e69425. 10.1371/journal.pone.0069425. 23869242 PMC3711813

[46] Corrigan RM, Abbott JC, Burhenne H, Kaever V, Gründling A. 2011. c-di-AMP is a new second messenger in *Staphylococcus aureus* with a role in controlling cell size and envelope stress. PLoS Pathog 7:e1002217. 10.1371/journal.ppat.1002217. 21909268 PMC3164647

[47] Griffiths JM, O'Neill AJ. 2012. Loss of function of the *gdpP* protein leads to joint β-lactam/glycopeptide tolerance in *Staphylococcus aureus*. Antimicrob Agents Chemother 56:579–581. 10.1128/AAC.05148-11. 21986827 PMC3256080

[48] Rismondo J, Gibhardt J, Rosenberg J, Kaever V, Halbedel S, Commichau FM. 2016. Phenotypes associated with the essential diadenylate cyclase CdaA and its potential regulator CdaR in the human pathogen *Listeria monocytogenes*. J Bacteriol 198:416–426. 10.1128/JB.00845-15. 26527648 PMC4719461

[49] Banerjee R, Gretes M, Harlem C, Basuino L, Chambers HF. 2010. A *mecA*-negative strain of methicillin-resistant *Staphylococcus aureus* with high-level β-lactam resistance contains mutations in three genes. Antimicrob Agents Chemother 54:4900–4902. 10.1128/AAC.00594-10. 20805396 PMC2976154

[50] Pozzi C, Waters EM, Rudkin JK, Schaeffer CR, Lohan AJ, Tong P, Loftus BJ, Pier GB, Fey PD, Massey RC, O'Gara JP. 2012. Methicillin resistance alters the biofilm phenotype and attenuates virulence in *Staphylococcus aureus* device-associated infections. PLoS Pathog 8:e1002626. 10.1371/journal.ppat.1002626. 22496652 PMC3320603

[51] Rao F, Ji Q, Soehano I, Liang ZX. 2011. Unusual heme-binding PAS domain from YybT family proteins. J Bacteriol 193:1543–1551. 10.1128/JB.01364-10. 21257773 PMC3067658

[52] Campos SS, Ibarra-Rodriguez JR, Barajas-Ornelas RC, Ramírez-Guadiana FH, Obregón-Herrera A, Setlow P, Pedraza-Reyes M. 2014. Interaction of apurinic/apyrimidinic endonucleases Nfo and ExoA with the DNA integrity scanning protein DisA in the processing of oxidative DNA damage during *Bacillus subtilis* spore outgrowth. J Bacteriol 196:568–578. 10.1128/JB.01259-13. 24244006 PMC3911150

[53] Rubin BE, Huynh TN, Welkie DG, Diamond S, Simkovsky R, Pierce EC, Taton A, Lowe LC, Lee JJ, Rifkin SA, Woodward JJ, Golden SS. 2018. High-throughput interaction screens illuminate the role of c-di-AMP in cyanobacterial nighttime survival. PLoS Genet 14:e1007301. 10.1371/journal.pgen.1007301. 29608558 PMC5897029

[54] Whiteley AT, Garelis NE, Peterson BN, Choi PH, Tong L, Woodward JJ, Portnoy DA. 2017. c-di-AMP modulates *Listeria monocytogenes* central metabolism to regulate growth, antibiotic resistance and osmoregulation. Mol Microbiol 104:212–233. 10.1111/mmi.13622. 28097715 PMC5391996

[55] Corrigan RM, Bowman L, Willis AR, Kaever V, Gründling A. 2015. Cross-talk between two nucleotide-signaling pathways in *Staphylococcus aureus*. J Biol Chem 290:5826–5839. 10.1074/jbc.M114.598300. 25575594 PMC4342491

[56] Whiteley AT, Pollock AJ, Portnoy DA. 2015. The PAMP c-di-AMP is essential for *Listeria monocytogenes* growth in rich but not minimal media due to a toxic increase in (p)ppGpp. [corrected]. Cell Host Microbe 17:788–798. 10.1016/j.chom.2015.05.006. 26028365 PMC4469362

[57] Peterson BN, Young MKM, Luo S, Wang J, Whiteley AT, Woodward JJ, Tong L, Wang JD, Portnoy DA. 2020. (p)ppGpp and c-di-AMP homeostasis Is controlled by CbpB in *Listeria monocytogenes*. mBio 11:e01625-20. 10.1128/mBio.01625-20. 32843560 PMC8549634

[58] Commichau FM, Gibhardt J, Halbedel S, Gundlach J, Stulke J. 2018. A delicate connection: c-di-AMP affects cell integrity by controlling osmolyte transport. Trends Microbiol 26:175–185. 10.1016/j.tim.2017.09.003. 28965724

[59] Gundlach J, Commichau FM, Stulke J. 2018. Perspective of ions and messengers: an intricate link between potassium, glutamate, and cyclic di-AMP. Curr Genet 64:191–195. 10.1007/s00294-017-0734-3. 28825218

[60] Chaudhuri RR, Allen AG, Owen PJ, Shalom G, Stone K, Harrison M, Burgis TA, Lockyer M, Garcia-Lara J, Foster SJ, Pleasance SJ, Peters SE, Maskell DJ, Charles IG. 2009. Comprehensive identification of essential *Staphylococcus aureus* genes using transposon-mediated differential hybridisation (TMDH). BMC Genomics 10:291. 10.1186/1471-2164-10-291. 19570206 PMC2721850

[61] Zeden MS, Schuster CF, Bowman L, Zhong Q, Williams HD, Gründling A. 2018. Cyclic di-adenosine monophosphate (c-di-AMP) is required for osmotic regulation in *Staphylococcus aureus* but dispensable for viability in anaerobic conditions. J Biol Chem 293:3180–3200. 10.1074/jbc.M117.818716. 29326168 PMC5836111

[62] Song JH, Ko KS, Lee JY, Baek JY, Oh WS, Yoon HS, Jeong JY, Chun J. 2005. Identification of essential genes in *Streptococcus pneumoniae* by allelic replacement mutagenesis. Mol Cells 19:365–374. 15995353

[63] Lluch-Senar M, Delgado J, Chen WH, Lloréns-Rico V, O'Reilly FJ, Wodke JA, Unal EB, Yus E, Martínez S, Nichols RJ, Ferrar T, Vivancos A, Schmeisky A, Stülke J, van Noort V, Gavin AC, Bork P, Serrano L. 2015. Defining a minimal cell: essentiality of small ORFs and ncRNAs in a genome-reduced bacterium. Mol Syst Biol 11:780. 10.15252/msb.20145558. 25609650 PMC4332154

[64] Ye M, Zhang JJ, Fang X, Lawlis GB, Troxell B, Zhou Y, Gomelsky M, Lou Y, Yang XF. 2014. DhhP, a cyclic di-AMP phosphodiesterase of *Borrelia burgdorferi*, is essential for cell growth and virulence. Infect Immun 82:1840–1849. 10.1128/IAI.00030-14. 24566626 PMC3993442

[65] Gundlach J, Mehne FM, Herzberg C, Kampf J, Valerius O, Kaever V, Stulke J. 2015. An essential poison: synthesis and degradation of cyclic di-AMP in *Bacillus subtilis*. J Bacteriol 197:3265–3274. 10.1128/JB.00564-15. 26240071 PMC4573722

[66] Blötz C, Treffon K, Kaever V, Schwede F, Hammer E, Stülke J. 2017. Identification of the components involved in cyclic di-AMP signaling in *Mycoplasma pneumoniae*. Front Microbiol 8:1328. 10.3389/fmicb.2017.01328. 28751888 PMC5508000

[67] Bai Y, Yang J, Eisele LE, Underwood AJ, Koestler BJ, Waters CM, Metzger DW, Bai G. 2013. Two DHH subfamily 1 proteins in *Streptococcus pneumoniae* possess cyclic di-AMP phosphodiesterase activity and affect bacterial growth and virulence. J Bacteriol 195:5123–5132. 10.1128/JB.00769-13. 24013631 PMC3811582

[68] Huynh TN, Luo S, Pensinger D, Sauer JD, Tong L, Woodward JJ. 2015. An HD-domain phosphodiesterase mediates cooperative hydrolysis of c-di-AMP to affect bacterial growth and virulence. Proc Natl Acad Sci U S A 112:E747–56. 10.1073/pnas.1416485112. 25583510 PMC4343097

[69] Du B, Ji W, An H, Shi Y, Huang Q, Cheng Y, Fu Q, Wang H, Yan Y, Sun J. 2014. Functional analysis of c-di-AMP phosphodiesterase, GdpP, in *Streptococcus suis* serotype 2. Microbiol Res 169:749–758. 10.1016/j.micres.2014.01.002. 24680501

[70] Yang J, Bai Y, Zhang Y, Gabrielle VD, Jin L, Bai G. 2014. Deletion of the cyclic di-AMP phosphodiesterase gene (*cnpB*) in *Mycobacterium tuberculosis* leads to reduced virulence in a mouse model of infection. Mol Microbiol 93:65–79. 10.1111/mmi.12641. 24806618 PMC4088933

[71] Hu J, Zhang G, Liang L, Lei C, Sun X. 2020. Increased excess intracellular cyclic di-AMP levels impair growth and virulence of *Bacillus anthracis*. J Bacteriol 202:e00653-19. 10.1128/JB.00653-19. 32071095 PMC7148140

[72] Peng X, Zhang Y, Bai G, Zhou X, Wu H. 2016. Cyclic di-AMP mediates biofilm formation. Mol Microbiol 99:945–959. 10.1111/mmi.13277. 26564551 PMC5003771

[73] Teh WK, Dramsi S, Tolker-Nielsen T, Yang L, Givskov M. 2019. Increased intracellular cyclic di-AMP levels sensitize *Streptococcus gallolyticus* subsp. *gallolyticus* to osmotic stress and reduce biofilm formation and adherence on intestinal cells. J Bacteriol 201:e00597-18. 10.1128/JB.00597-18. 30617242 PMC6398277

[74] Konno H, Yoshida Y, Nagano K, Takebe J, Hasegawa Y. 2018. Biological and biochemical roles of two distinct cyclic dimeric adenosine 3',5'-monophosphate-associated phosphodiesterases in *Streptococcus mutans*. Front Microbiol 9:2347. 10.3389/fmicb.2018.02347. 30319597 PMC6170606

[75] Townsley L, Yannarell SM, Huynh TN, Woodward JJ, Shank EA. 2018. Cyclic di-AMP acts as an extracellular signal that impacts *Bacillus subtilis* biofilm formation and plant attachment. mBio 9:e00341-18. 10.1128/mBio.00341-18. 29588402 PMC5874923

[76] Gundlach J, Rath H, Herzberg C, Mäder U, Stülke J. 2016. Second messenger signaling in *Bacillus subtilis*: accumulation of cyclic di-AMP inhibits biofilm formation. Front Microbiol 7:804. 10.3389/fmicb.2016.00804. 27252699 PMC4879592

[77] Fraser CM, Casjens S, Huang WM, Sutton GG, Clayton R, Lathigra R, White O, Ketchum KA, Dodson R, Hickey EK, Gwinn M, Dougherty B, Tomb JF, Fleischmann RD, Richardson D, Peterson J, Kerlavage AR, Quackenbush J, Salzberg S, Hanson M, van Vugt R, Palmer N, Adams MD, Gocayne J, Weidman J, Utterback T, Watthey L, McDonald L, Artiach P, Bowman C, Garland S, Fuji C, Cotton MD, Horst K, Roberts K, Hatch B, Smith HO, Venter JC. 1997. Genomic sequence of a Lyme disease spirochaete, *Borrelia burgdorferi*. Nature 390:580–586. 10.1038/37551. 9403685

[78] Savage CR, Arnold WK, Gjevre-Nail A, Koestler BJ, Bruger EL, Barker JR, Waters CM, Stevenson B. 2015. Intracellular concentrations of *Borrelia burgdorferi* cyclic di-AMP are not changed by altered expression of the CdaA synthase. PLoS One 10:e0125440. 10.1371/journal.pone.0125440. 25906393 PMC4408052

[79] Gupta RS. 2019. Distinction between *Borrelia* and *Borreliella* is more robustly supported by molecular and phenotypic characteristics than all other neighbouring prokaryotic genera: response to Margos' et al. “The genus *Borrelia* reloaded” (PLoS ONE 13(12): e0208432). PLoS One 14:e0221397. 10.1371/journal.pone.0221397. 31454394 PMC6711536

[80] Steere AC, Strle F, Wormser GP, Hu LT, Branda JA, Hovius JW, Li X, Mead PS. 2016. Lyme borreliosis. Nat Rev Dis Primers 2:16090. 10.1038/nrdp.2016.90. 27976670 PMC5539539

[81] Adeolu M, Gupta RS. 2014. A phylogenomic and molecular marker based proposal for the division of the genus *Borrelia* into two genera: the emended genus *Borrelia* containing only the members of the relapsing fever *Borrelia*, and the genus *Borreliella* gen. nov. containing the members of the Lyme disease *Borrelia* (*Borrelia burgdorferi* sensu lato complex). Antonie Van Leeuwenhoek 105:1049–1072. 10.1007/s10482-014-0164-x. 24744012

[82] Witte G, Hartung S, Büttner K, Hopfner KP. 2008. Structural biochemistry of a bacterial checkpoint protein reveals diadenylate cyclase activity regulated by DNA recombination intermediates. Mol Cell 30:167–178. 10.1016/j.molcel.2008.02.020. 18439896

[83] Woodward JJ, Iavarone AT, Portnoy DA. 2010. c-di-AMP secreted by intracellular *Listeria monocytogenes* activates a host type I interferon response. Science 328:1703–1705. 10.1126/science.1189801. 20508090 PMC3156580

[84] Lu S, Wang J, Chitsaz F, Derbyshire MK, Geer RC, Gonzales NR, Gwadz M, Hurwitz DI, Marchler GH, Song JS, Thanki N, Yamashita RA, Yang M, Zhang D, Zheng C, Lanczycki CJ, Marchler-Bauer A. 2020. CDD/SPARCLE: the conserved domain database in 2020. Nucleic Acids Res 48:D265–D268. 10.1093/nar/gkz991. 31777944 PMC6943070

[85] Sonnhammer EL, von Heijne G, Krogh A. 1998. A hidden Markov model for predicting transmembrane helices in protein sequences. Proc Int Conf Intell Syst Mol Biol 6:175–182. 9783223

[86] Krogh A, Larsson B, von Heijne G, Sonnhammer EL. 2001. Predicting transmembrane protein topology with a hidden Markov model: application to complete genomes. J Mol Biol 305:567–580. 10.1006/jmbi.2000.4315. 11152613

[87] Möller S, Croning MD, Apweiler R. 2001. Evaluation of methods for the prediction of membrane spanning regions. Bioinformatics 17:646–653. 10.1093/bioinformatics/17.7.646. 11448883

[88] Simm D, Hatje K, Kollmar M. 2015. Waggawagga: comparative visualization of coiled-coil predictions and detection of stable single α-helices (SAH domains). Bioinformatics 31:767–769. 10.1093/bioinformatics/btu700. 25338722

[89] Jackson-Litteken CD, Zalud AK, Ratliff CT, Latham JI, Bourret TJ, Lopez JE, Blevins JS. 2019. Assessing the contribution of an HtrA family serine protease during *Borrelia turicatae* mammalian infection. Front Cell Infect Microbiol 9:290. 10.3389/fcimb.2019.00290. 31456953 PMC6700303

[90] Rosenberg J, Dickmanns A, Neumann P, Gunka K, Arens J, Kaever V, Stülke J, Ficner R, Commichau FM. 2015. Structural and biochemical analysis of the essential diadenylate cyclase CdaA from *Listeria monocytogenes*. J Biol Chem 290:6596–6606. 10.1074/jbc.M114.630418. 25605729 PMC4358292

[91] Maguire ME. 2006. Magnesium transporters: properties, regulation and structure. Front Biosci 11:3149–3163. 10.2741/2039. 16720382

[92] Payandeh J, Pfoh R, Pai EF. 2013. The structure and regulation of magnesium selective ion channels. Biochim Biophys Acta 1828:2778–2792. 10.1016/j.bbamem.2013.08.002. 23954807

[93] Johnson JW, Fisher JF, Mobashery S. 2013. Bacterial cell-wall recycling. Ann N Y Acad Sci 1277:54–75. 10.1111/j.1749-6632.2012.06813.x. 23163477 PMC3556187

[94] Hantke K. 2005. Bacterial zinc uptake and regulators. Curr Opin Microbiol 8:196–202. 10.1016/j.mib.2005.02.001. 15802252

[95] Pittman JK. 2005. Managing the manganese: molecular mechanisms of manganese transport and homeostasis. New Phytol 167:733–742. 10.1111/j.1469-8137.2005.01453.x. 16101910

[96] Groshong AM, Dey A, Bezsonova I, Caimano MJ, Radolf JD. 2017. Peptide uptake Is essential for *Borrelia burgdorferi* viability and involves structural and regulatory complexity of its oligopeptide transporter. mBio 8:e02047-17. 10.1128/mBio.02047-17. 29259089 PMC5736914

[97] Duval M, Korepanov A, Fuchsbauer O, Fechter P, Haller A, Fabbretti A, Choulier L, Micura R, Klaholz BP, Romby P, Springer M, Marzi S. 2013. *Escherichia coli* ribosomal protein S1 unfolds structured mRNAs onto the ribosome for active translation initiation. PLoS Biol 11:e1001731. 10.1371/journal.pbio.1001731. 24339747 PMC3858243

[98] Schwan TG, Battisti JM, Porcella SF, Raffel SJ, Schrumpf ME, Fischer ER, Carroll JA, Stewart PE, Rosa P, Somerville GA. 2003. Glycerol-3-phosphate acquisition in spirochetes: distribution and biological activity of glycerophosphodiester phosphodiesterase (GlpQ) among *Borrelia* species. JB 185:1346–1356. 10.1128/JB.185.4.1346-1356.2003. PMC142843 12562805

[99] Latham JI, Blevins JS. 2018. Generation of conditional mutants in *Borrelia burgdorferi*. Methods Mol Biol 1690:225–239. 10.1007/978-1-4939-7383-5_17. 29032548

[100] Groshong AM, Gibbons NE, Yang XF, Blevins JS. 2012. Rrp2, a prokaryotic enhancer-like binding protein, is essential for viability of *Borrelia burgdorferi*. J Bacteriol 194:3336–3342. 10.1128/JB.00253-12. 22544267 PMC3434732

[101] Caimano MJ, Eggers CH, Hazlett KR, Radolf JD. 2004. RpoS is not central to the general stress response in *Borrelia burgdorferi* but does control expression of one or more essential virulence determinants. Infect Immun 72:6433–6445. 10.1128/IAI.72.11.6433-6445.2004. 15501774 PMC523033

[102] Hubner A, Yang X, Nolen DM, Popova TG, Cabello FC, Norgard MV. 2001. Expression of *Borrelia burgdorferi* OspC and DbpA is controlled by a RpoN-RpoS regulatory pathway. Proc Natl Acad Sci U S A 98:12724–12729. 10.1073/pnas.231442498. 11675503 PMC60121

[103] Caimano MJ, Iyer R, Eggers CH, Gonzalez C, Morton EA, Gilbert MA, Schwartz I, Radolf JD. 2007. Analysis of the RpoS regulon in *Borrelia burgdorferi* in response to mammalian host signals provides insight into RpoS function during the enzootic cycle. Mol Microbiol 65:1193–1217. 10.1111/j.1365-2958.2007.05860.x. 17645733 PMC2967192

[104] Hyde JA, Shaw DK, Smith Iii R, Trzeciakowski JP, Skare JT. 2009. The BosR regulatory protein of *Borrelia burgdorferi* interfaces with the RpoS regulatory pathway and modulates both the oxidative stress response and pathogenic properties of the Lyme disease spirochete. Mol Microbiol 74:1344–1355. 10.1111/j.1365-2958.2009.06951.x. 19906179 PMC2805275

[105] Hyde JA, Shaw DK, Smith R, III, Trzeciakowski JP, Skare JT. 2010. Characterization of a conditional *bosR* mutant in *Borrelia burgdorferi*. Infect Immun 78:265–274. 10.1128/IAI.01018-09. 19858309 PMC2798208

[106] Ouyang Z, Kumar M, Kariu T, Haq S, Goldberg M, Pal U, Norgard MV. 2009. BosR (BB0647) governs virulence expression in *Borrelia burgdorferi*. Mol Microbiol 74:1331–1343. 10.1111/j.1365-2958.2009.06945.x. 19889086 PMC2831293

[107] Ouyang Z, Deka RK, Norgard MV. 2011. BosR (BB0647) controls the RpoN-RpoS regulatory pathway and virulence expression in *Borrelia burgdorferi* by a novel DNA-binding mechanism. PLoS Pathog 7:e1001272. 10.1371/journal.ppat.1001272. 21347346 PMC3037356

[108] Huynh TN, Choi PH, Sureka K, Ledvina HE, Campillo J, Tong L, Woodward JJ. 2016. Cyclic di-AMP targets the cystathionine beta-synthase domain of the osmolyte transporter OpuC. Mol Microbiol 102:233–243. 10.1111/mmi.13456. 27378384 PMC5118871

[109] Hunfeld KP, Kraiczy P, Wichelhaus TA, Schafer V, Brade V. 2000. New colorimetric microdilution method for in vitro susceptibility testing of *Borrelia burgdorferi* against antimicrobial substances. Eur J Clin Microbiol Infect Dis 19:27–32. 10.1007/s100960050005. 10706176

[110] Koetsveld J, Draga ROP, Wagemakers A, Manger A, Oei A, Visser CE, Hovius JW. 2017. In vitro susceptibility of the relapsing-fever spirochete *Borrelia miyamotoi* to antimicrobial agents. Antimicrob Agents Chemother 61:e00535-17. 10.1128/AAC.00535-17. 28674060 PMC5571331

[111] Jutras BL, Chenail AM, Stevenson B. 2013. Changes in bacterial growth rate govern expression of the *Borrelia burgdorferi* OspC and Erp infection-associated surface proteins. J Bacteriol 195:757–764. 10.1128/JB.01956-12. 23222718 PMC3562092

[112] Graham DE, Groshong AM, Jackson-Litteken CD, Moore BP, Caimano MJ, Blevins JS. 2020. The BB0345 hypothetical protein of *Borrelia burgdorferi* is essential for mammalian infection. Infect Immun 88:e00472-20. 10.1128/IAI.00472-20. 32928963 PMC7671889

[113] Bontemps-Gallo S, Lawrence K, Gherardini FC. 2016. Two different virulence-related regulatory pathways in *Borrelia burgdorferi* are directly affected by osmotic fluxes in the blood meal of feeding *Ixodes* ticks. PLoS Pathog 12:e1005791. 10.1371/journal.ppat.1005791. 27525653 PMC4985143

[114] Troxell B, Zhang JJ, Bourret TJ, Zeng MY, Blum J, Gherardini F, Hassan HM, Yang XF. 2014. Pyruvate protects pathogenic spirochetes from H_2_O_2_ killing. PLoS One 9:e84625. 10.1371/journal.pone.0084625. 24392147 PMC3879313

[115] Bourret TJ, Boyle WK, Zalud AK, Valenzuela JG, Oliveira F, Lopez JE. 2019. The relapsing fever spirochete *Borrelia turicatae* persists in the highly oxidative environment of its soft-bodied tick vector. Cell Microbiol 21:e12987. 10.1111/cmi.12987. 30489694 PMC6454574

[116] Talagrand-Reboul E, Boyer PH, Bergstrom S, Vial L, Boulanger N. 2018. Relapsing fevers: neglected tick-borne diseases. Front Cell Infect Microbiol 8:98. 10.3389/fcimb.2018.00098. 29670860 PMC5893795

[117] Dworkin MS, Shoemaker PC, Fritz CL, Dowell ME, Anderson DE, Jr. 2002. The epidemiology of tick-borne relapsing fever in the United States. Am J Trop Med Hyg 66:753–758. 10.4269/ajtmh.2002.66.753. 12224586

[118] Alugupalli KR, Gerstein RM, Chen J, Szomolanyi-Tsuda E, Woodland RT, Leong JM. 2003. The resolution of relapsing fever borreliosis requires IgM and is concurrent with expansion of B1b lymphocytes. J Immunol 170:3819–3827. 10.4049/jimmunol.170.7.3819. 12646649

[119] Connolly SE, Benach JL. 2001. Cutting edge: the spirochetemia of murine relapsing fever is cleared by complement-independent bactericidal antibodies. J Immunol 167:3029–3032. 10.4049/jimmunol.167.6.3029. 11544285

[120] Cadavid D, Londono D. 2009. Understanding tropism and immunopathological mechanisms of relapsing fever spirochaetes. Clin Microbiol Infect 15:415–421. 10.1111/j.1469-0691.2009.02785.x. 19489924 PMC2782903

[121] Crowder CD, Ghalyanchi Langeroudi A, Shojaee Estabragh A, Lewis ERG, Marcsisin RA, Barbour AG. 2016. Pathogen and host response dynamics in a mouse model of *Borrelia hermsii* relapsing fever. Vet Sci 3:19. 10.3390/vetsci3030019. PMC5606581 29056727

[122] Boyle WK, Wilder HK, Lawrence AM, Lopez JE. 2014. Transmission dynamics of *Borrelia turicatae* from the arthropod vector. PLoS Negl Trop Dis 8:e2767. 10.1371/journal.pntd.0002767. 24699275 PMC3974661

[123] Mehne FM, Gunka K, Eilers H, Herzberg C, Kaever V, Stulke J. 2013. Cyclic di-AMP homeostasis in *Bacillus subtilis*: both lack and high level accumulation of the nucleotide are detrimental for cell growth. J Biol Chem 288:2004–2017. 10.1074/jbc.M112.395491. 23192352 PMC3548507

[124] Gibhardt J, Heidemann JL, Bremenkamp R, Rosenberg J, Seifert R, Kaever V, Ficner R, Commichau FM. 2020. An extracytoplasmic protein and a moonlighting enzyme modulate synthesis of c-di-AMP in *Listeria monocytogenes*. Environ Microbiol 22:2771–2791. 10.1111/1462-2920.15008. 32250026

[125] Pham TH, Liang ZX, Marcellin E, Turner MS. 2016. Replenishing the cyclic-di-AMP pool: regulation of diadenylate cyclase activity in bacteria. Curr Genet 62:731–738. 10.1007/s00294-016-0600-8. 27074767

[126] Zhu Y, Pham TH, Nhiep TH, Vu NM, Marcellin E, Chakrabortti A, Wang Y, Waanders J, Lo R, Huston WM, Bansal N, Nielsen LK, Liang ZX, Turner MS. 2016. Cyclic-di-AMP synthesis by the diadenylate cyclase CdaA is modulated by the peptidoglycan biosynthesis enzyme GlmM in *Lactococcus lactis*. Mol Microbiol 99:1015–1027. 10.1111/mmi.13281. 26585449

[127] Elovson J, Vagelos PR. 1968. Acyl carrier protein. X. Acyl carrier protein synthetase. J Biol Chem 243:3603–3611. 10.1016/S0021-9258(19)34183-3. 4872726

[128] Gundlach J, Herzberg C, Kaever V, Gunka K, Hoffmann T, Weiß M, Gibhardt J, Thürmer A, Hertel D, Daniel R, Bremer E, Commichau FM, Stülke J. 2017. Control of potassium homeostasis is an essential function of the second messenger cyclic di-AMP in *Bacillus subtilis*. Sci Signal 10:eaal3011. 10.1126/scisignal.aal3011. 28420751

[129] Devaux L, Sleiman D, Mazzuoli MV, Gominet M, Lanotte P, Trieu-Cuot P, Kaminski PA, Firon A. 2018. Cyclic di-AMP regulation of osmotic homeostasis is essential in group B *Streptococcus*. PLoS Genet 14:e1007342. 10.1371/journal.pgen.1007342. 29659565 PMC5919688

[130] Gundlach J, Krüger L, Herzberg C, Turdiev A, Poehlein A, Tascón I, Weiss M, Hertel D, Daniel R, Hänelt I, Lee VT, Stülke J. 2019. Sustained sensing in potassium homeostasis: cyclic di-AMP controls potassium uptake by KimA at the levels of expression and activity. J Biol Chem 294:9605–9614. 10.1074/jbc.RA119.008774. 31061098 PMC6579464

[131] Mehne FM, Schröder-Tittmann K, Eijlander RT, Herzberg C, Hewitt L, Kaever V, Lewis RJ, Kuipers OP, Tittmann K, Stülke J. 2014. Control of the diadenylate cyclase CdaS in *Bacillus subtilis*: an autoinhibitory domain limits cyclic di-AMP production. J Biol Chem 289:21098–21107. 10.1074/jbc.M114.562066. 24939848 PMC4110313

[132] Stülke J, Krüger L. 2020. Cyclic di-AMP signaling in bacteria. Annu Rev Microbiol 74:159–179. 10.1146/annurev-micro-020518-115943. 32603625

[133] Commichau FM, Dickmanns A, Gundlach J, Ficner R, Stulke J. 2015. A jack of all trades: the multiple roles of the unique essential second messenger cyclic di-AMP. Mol Microbiol 97:189–204. 10.1111/mmi.13026. 25869574

[134] Bennett BD, Redford KE, Gralnick JA. 2018. MgtE homolog FicI acts as a secondary ferrous iron importer in *Shewanella oneidensis* strain MR-1. Appl Environ Microbiol 84:e01245-17. 10.1128/AEM.01245-17. 29330185 PMC5835737

[135] Maria-Rosario A, Davidson I, Debra M, Verheul A, Abee T, Booth IR. 1995. The role of peptide metabolism in the growth of *Listeria monocytogenes* ATCC 23074 at high osmolarity. Microbiology (Reading) 141:41–49. 10.1099/00221287-141-1-41. 7894718

[136] Rodriguez MB, Costa SOP. 1999. Spontaneous kanamycin-resistant *Escherichia coli* mutant with altered periplasmic oligopeptide permease protein (OppA) and impermeability to aminoglycosides. Rev Microbiol 30:153–156. 10.1590/S0001-37141999000200013.

[137] Nakamatsu EH, Fujihira E, Ferreira RC, Balan A, Costa SO, Ferreira LC. 2007. Oligopeptide uptake and aminoglycoside resistance in *Escherichia coli* K12. FEMS Microbiol Lett 269:229–233. 10.1111/j.1574-6968.2007.00634.x. 17250759

[138] Acosta MBR, Ferreira RCC, Padilla G, Ferreira LCS, Costa SOP. 2000. Altered expression of oligopeptide-binding protein (OppA) and aminoglycoside resistance in laboratory and clinical *Escherichia coli* strains. J Med Microbiol 49:409–413. 10.1099/0022-1317-49-5-409. 10798552

[139] Fisher MA, Grimm D, Henion AK, Elias AF, Stewart PE, Rosa PA, Gherardini FC. 2005. *Borrelia burgdorferi* sigma54 is required for mammalian infection and vector transmission but not for tick colonization. Proc Natl Acad Sci U S A 102:5162–5167. 10.1073/pnas.0408536102. 15743918 PMC555983

[140] Ouyang Z, Blevins JS, Norgard MV. 2008. Transcriptional interplay among the regulators Rrp2, RpoN and RpoS in *Borrelia burgdorferi*. Microbiology (Reading) 154:2641–2658. 10.1099/mic.0.2008/019992-0. 18757798

[141] Elbir H, Abi-Rached L, Pontarotti P, Yoosuf N, Drancourt M. 2014. African relapsing fever borreliae genomospecies revealed by comparative genomics. Front Public Health 2:43. 10.3389/fpubh.2014.00043. 25229054 PMC4157404

[142] Sureka K, Choi PH, Precit M, Delince M, Pensinger DA, Huynh TN, Jurado AR, Goo YA, Sadilek M, Iavarone AT, Sauer JD, Tong L, Woodward JJ. 2014. The cyclic dinucleotide c-di-AMP is an allosteric regulator of metabolic enzyme function. Cell 158:1389–1401. 10.1016/j.cell.2014.07.046. 25215494 PMC4166403

[143] Choi PH, Vu TMN, Pham HT, Woodward JJ, Turner MS, Tong L. 2017. Structural and functional studies of pyruvate carboxylase regulation by cyclic di-AMP in lactic acid bacteria. Proc Natl Acad Sci U S A 114:E7226–E7235. 10.1073/pnas.1704756114. 28808024 PMC5584425

[144] Corona A, Schwartz I. 2015. *Borrelia burgdorferi*: carbon metabolism and the tick-mammal enzootic cycle. Microbiol Spectr 3. 10.1128/microbiolspec.MBP-0011-2014. PMC7942402 26185064

[145] von Lackum K, Stevenson B. 2005. Carbohydrate utilization by the Lyme borreliosis spirochete, *Borrelia burgdorferi*. FEMS Microbiol Lett 243:173–179. 10.1016/j.femsle.2004.12.002. 15668016

[146] Boylan JA, Hummel CS, Benoit S, Garcia-Lara J, Treglown-Downey J, Crane EJ, Gherardini FC. 2006. *Borrelia burgdorferi bb0728* encodes a coenzyme A disulphide reductase whose function suggests a role in intracellular redox and the oxidative stress response. Mol Microbiol 59:475–486. 10.1111/j.1365-2958.2005.04963.x. 16390443

[147] Eggers CH, Caimano MJ, Malizia RA, Kariu T, Cusack B, Desrosiers DC, Hazlett KR, Claiborne A, Pal U, Radolf JD. 2011. The coenzyme A disulphide reductase of *Borrelia burgdorferi* is important for rapid growth throughout the enzootic cycle and essential for infection of the mammalian host. Mol Microbiol 82:679–697. 10.1111/j.1365-2958.2011.07845.x. 21923763 PMC3226778

[148] Wolfe AJ. 2005. The acetate switch. Microbiol Mol Biol Rev 69:12–50. 10.1128/MMBR.69.1.12-50.2005. 15755952 PMC1082793

[149] Taylor J, Moore G, Cheek J. 1991. Outbreak of relapsing fever masquerading as Lyme borreliosis. Abstr. 30th Intersci Conf Antimicrob Agents Chemother. American Society for Microbiology Press, Washington, DC.

[150] Schwan TG, Raffel SJ, Schrumpf ME, Policastro PF, Rawlings JA, Lane RS, Breitschwerdt EB, Porcella SF. 2005. Phylogenetic analysis of the spirochetes *Borrelia parkeri* and *Borrelia turicatae* and the potential for tick-borne relapsing fever in Florida. J Clin Microbiol 43:3851–3859. 10.1128/JCM.43.8.3851-3859.2005. 16081922 PMC1233929

[151] Barbour AG. 1984. Isolation and cultivation of Lyme disease spirochetes. Yale J Biol Med 57:521–525. 6393604 PMC2589996

[152] Battisti JM, Raffel SJ, Schwan TG. 2008. A system for site-specific genetic manipulation of the relapsing fever spirochete *Borrelia hermsii*. Methods Mol Biol 431:69–84. 10.1007/978-1-60327-032-8_6. 18287748

[153] Stewart PE, Thalken R, Bono JL, Rosa P. 2001. Isolation of a circular plasmid region sufficient for autonomous replication and transformation of infectious *Borrelia burgdorferi*. Mol Microbiol 39:714–721. 10.1046/j.1365-2958.2001.02256.x. 11169111

[154] Blevins JS, Revel AT, Smith AH, Bachlani GN, Norgard MV. 2007. Adaptation of a luciferase gene reporter and *lac* expression system to *Borrelia burgdorferi*. Appl Environ Microbiol 73:1501–1513. 10.1128/AEM.02454-06. 17220265 PMC1828772

[155] Bono JL, Elias AF, Kupko JJ, III, Stevenson B, Tilly K, Rosa P. 2000. Efficient targeted mutagenesis in *Borrelia burgdorferi*. J Bacteriol 182:2445–2452. 10.1128/jb.182.9.2445-2452.2000. 10762244 PMC111306

[156] Lopez JE, Wilder HK, Hargrove R, Brooks CP, Peterson KE, Beare PA, Sturdevant DE, Nagarajan V, Raffel SJ, Schwan TG. 2013. Development of genetic system to inactivate a *Borrelia turicatae* surface protein selectively produced within the salivary glands of the arthropod vector. PLoS Negl Trop Dis 7:e2514. 10.1371/journal.pntd.0002514. 24205425 PMC3814808

[157] Novy R, Drott D, Yaeger K, Mierendorf R. 2001. Overcoming the codon bias of *E. coli* for enhanced protein expression. inNovations 12:1–3.

[158] Groshong AM, Fortune DE, Moore BP, Spencer HJ, Skinner RA, Bellamy WT, Blevins JS. 2014. BB0238, a presumed tetratricopeptide repeat-containing protein, is required during *Borrelia burgdorferi* mammalian infection. Infect Immun 82:4292–4306. 10.1128/IAI.01977-14. 25069985 PMC4187884

[159] Blevins JS, Hagman KE, Norgard MV. 2008. Assessment of decorin-binding protein A to the infectivity of *Borrelia burgdorferi* in the murine models of needle and tick infection. BMC Microbiol 8:82. 10.1186/1471-2180-8-82. 18507835 PMC2430964

[160] Bernstein JA, Khodursky AB, Lin PH, Lin-Chao S, Cohen SN. 2002. Global analysis of mRNA decay and abundance in *Escherichia coli* at single-gene resolution using two-color fluorescent DNA microarrays. Proc Natl Acad Sci U S A 99:9697–9702. 10.1073/pnas.112318199. 12119387 PMC124983

[161] Chen S, Zhou Y, Chen Y, Gu J. 2018. fastp: an ultra-fast all-in-one FASTQ preprocessor. Bioinformatics 34:i884–i890. 10.1093/bioinformatics/bty560. 30423086 PMC6129281

[162] Seemann T. 2013. Snippy: fast bacterial variant calling from NGS reads. https://github.com/tseemann/snippy.

[163] Jorgenson MA, Bryant JC. 2021. A genetic screen to identify factors affected by undecaprenyl phosphate recycling uncovers novel connections to morphogenesis in *Escherichia coli*. Mol Microbiol 115:191–207. 10.1111/mmi.14609. 32979869 PMC10568968

[164] Livak KJ, Schmittgen TD. 2001. Analysis of relative gene expression data using real-time quantitative PCR and the 2(-delta C(T)) method. Methods 25:402–408. 10.1006/meth.2001.1262. 11846609

[165] Revel AT, Blevins JS, Almazan C, Neil L, Kocan KM, de la Fuente J, Hagman KE, Norgard MV. 2005. *bptA* (*bbe16*) is essential for the persistence of the Lyme disease spirochete, *Borrelia burgdorferi*, in its natural tick vector. Proc Natl Acad Sci U S A 102:6972–6977. 10.1073/pnas.0502565102. 15860579 PMC1100799

